# Reconsidering non-traditional export agriculture and household food security: A case study in rural Guatemala

**DOI:** 10.1371/journal.pone.0198113

**Published:** 2018-05-24

**Authors:** Josée Méthot, Elena M. Bennett

**Affiliations:** Department of Natural Resource Sciences and McGill School of Environment, McGill University, Ste-Anne-de-Bellevue, Quebec, Canada; Institut de recherche pour le developpement, FRANCE

## Abstract

As the production of non-traditional export (NTX) crops by smallholder households in developing countries expands, there is a compelling need to understand the potential effects of this type of agricultural production on household food security and nutrition. We use two household surveys with a sample of 52 households, interviews, and focus groups to examine whether smallholder farmers who produce broccoli for export in a rural Guatemalan community have different household food security than farmers in the same community who are still growing traditional maize and bean crops. We explore and compare the food security status of broccoli farmers (adopters) and traditional farmers (non-adopters) across four dimensions of food security: availability, access, utilization, and stability. Adopters earned significantly more income (40%) than non-adopters, but higher incomes did not coincide with improvements in food availability, food access, or food utilization. Results indicate that adopters and non-adopters alike struggle with access to food, while the intensity of broccoli production may be undermining the ability of local agricultural systems to naturally control pests and regulate nutrients. More systematic approaches to food security assessment, especially those that consider all four dimensions of food security, are needed to better target interventions designed to alleviate food insecurity among rural smallholders.

## Introduction

The increased commercialization of agriculture and diversification into non-traditional export crops (NTXs) by smallholder farmers has been touted as a growth-oriented strategy to reduce rural poverty and combat food insecurity [1]. NTXs include high-value, labour intensive fruits and vegetables that are not part of the customary diet of a local farming population, and are not traditionally farmed for export in a given country [2,3]. Global production of NTXs is booming in response to policies aimed at using NTX production to combat poverty and food insecurity: between 1992 and 2001, the worldwide trade in non-traditional vegetables rose sharply, from 7.6 million tonnes in 1992 to 13.9 million tonnes in 2001 [4]. By 2001, 63% of these exports came from developing countries and this share is growing quickly, driven by a recent upsurge in production in Central America and the Caribbean [4,5].

Much of the literature on NTXs focuses on their potential to improve national economic performance [6,7,8,9,10]. At this scale, the increased commercialization of agriculture and the diversification into high-value NTXs is promoted as a viable strategy for developing countries to stimulate growth in the agricultural sector, reduce unemployment, and stabilize or repay foreign debt [5]. National economic growth, however, is only one part of the development promise of NTXs; export promotion policies also propose to reduce rural poverty [11] and combat rural food insecurity [1]. NTXs have been heralded as a way for smallholder farmers to work their own lands, integrate into international markets, and increase household income through the sale of specialty export crops [6,12]. Altogether, these purported cross-scale benefits have helped frame NTX production as a ‘win-win’ strategy for development in that it improves the incomes and livelihoods of smallholders while simultaneously boosting local, national, and international economies [6,8]. In the push for global development, there is a tendency to assume that income poverty reduction is synonymous with hunger reduction and improvements in nutrition, despite evidence suggesting that these relationships are not so straightforward [13,14].

As smallholders in developing countries shift into the NTX sector, there is a compelling need to understand whether or not farming NTXs is likely to improve the household food security of smallholders. Previous analyses of the effects of NTXs on food security in real communities highlight mixed results [15,16,17]. In part, this may be because these quantitative studies, which mostly predate significant conceptual and methodological advances in food security research, assess food security too narrowly by today’s standards. Notably, we now recognize that food security includes four dimensions: food *availability*, food *access*, food *utilization*, and food system *stability* [18]. Achieving food security requires that that the *availability* of food is sufficient, that there is adequate economic and social *access* to food supplies through the market or through other means, and that the *utilization* of food supplies is sufficient to meet the specific dietary and nutritional needs of individuals [18]. Moreover, each of these factors must be *stable through time* in order to support food security in the long-term [19,20]. To date, however, as far as we know, no research has systematically explored the influence of NTX production on smallholders across all four dimensions of food security.

Studies that specifically examine the links between NTX production and one or more dimensions of food security indicate that NTX production can increase household income and thus positively influence food access, however the implications for the other three dimensions are less clear (Table 1). Some studies show that NTX production has a positive effect on a given dimension, whereas others show a neutral or negative effect. The differences in results may in part reflect the heterogeneity of social-ecological contexts of these studies, as well as the diversity of indicators employed.

**Table 1:** Key studies exploring the food security implications of NTX production across the four dimensions of food security: Availability, access, utilization, and stability.

Study	Country	Crop	*Effects on*			
			Availability	Access	Utilization	Stability
Carletto et al. (2011) [3]	Guatemala	Snow peas	n.a.	Positive *(Food expenditures)*	n.a.	Negative *(Crop yield over 20 years)*
von Braun et al. (1989) [16]	Guatemala	Snow pea	Positive *(Staple production; caloric intake)*	Positive *(Income; food expenditures)*	Neutral to slightly positive *(Anthropometry)*	n.a.
Immink and Alarcon (1993) [17]	Guatemala	Broccoli, cauliflower, cabbage	Negative *(Staple production)* Neutral *(Dietary energy and protein intake)*	Positive *(Income)*	n.a.	n.a.
Immink and Alarcon (1991) [21]	Guatemala	Broccoli, cauliflower, cabbage	Neutral *(Dietary energy intake)*	Positive *(Income)*	Neutral *(Micronutrient intake)*	n.a.
Katz (1994) [22]	Guatemala	Snow peas; Broccoli	Neutral *(Staple production)*	Positive *(Income)*	n.a.	n.a.
Govereh and Jayne (2003) [23]	Zimbabwe	Cotton	Neutral *(Staple production)*	Positive *(Income)*	n.a	n.a.
Hamilton and Fischer (2003) [24]	Guatemala	Snow peas, broccoli	n.a.	Positive *(Income)*	Positive *(Perception of nutritional improvement by mothers)*	n.a
Webb et al. (2016) [25]	Guatemala	Green beans; peas; broccoli; blackberries	Negative *(Land dedicated to subsistence production)*	*Negative* *(Food expenditures)*	n.a.	n.a.

The effects of NTX adoption on each of the four dimensions are classified as positive (shaded light grey), neutral (unshaded), or negative (shaded dark grey), and the indicator(s) used to capture each dimension are italicized.

Generally speaking, efforts to measure food security and target interventions have been thwarted by the difficult conceptual and technical challenges that are inherent in capturing a multi-dimensional problem like food security [26]. Researchers and practitioners often cherry-pick indicators, or rely on a single indicator to capture the food security concept as a whole [26], all the while failing to account for the fact that different food security indicators are needed to assess the different dimensions of food security [27]. To avoid these common pitfalls, Coates (2013) suggests taking a systematic, deconstructed approach to food security measurement, where complementary indicators are selected to independently assess different dimensions of food security [26].

Heeding this advice, we systematically assessed the food security status of households that farm a non-traditional export crop, broccoli (*Brassica oleracea*), in a small rural community in Guatemala. We ask: does the food security status of households that farm NTX crops in the Guatemalan community of Chilascó differ from those that farm traditional crops across the four dimensions of household food security? Using a case study approach and mixed methods, we compared levels of food security across households that have adopted broccoli production as a livelihood strategy (adopters) relative to households who do not farm broccoli, and instead farm traditional corn, bean, or other secondary crops (non-adopters). While the NTX and policy literature suggests that NTX farmers might have higher food security than non-adopters, we hypothesized that the food security status of adopters versus non-adopters would depend on the dimension of food security considered. See Table 2 for an expansion of this conceptual framework, including key definitions, study hypotheses, and literature related to each of the four dimensions of food security. We employed indicators of food availability, food access, and food utilization, and introduce the idea of using indicators of ecosystem services as an entry-point to understanding the dimension of food stability at the household level. By systematically assessing the household food security of smallholders across all four dimensions of food security–including the oft-neglected dimension of food system stability–we expect to contribute to food security research, policy, and practice by improving the design of future assessments and interventions.

**Table 2:** The four guiding research hypotheses for this study in relation to the four dimensions of food security.

Dimension and Definition	Hypothesis	Rationale
Availability refers to the “supply side” of food security, specifically the availability of sufficient quantities of food, as supplied by domestic production, imports, or food aid [28].	The availability of staple crops (corn and bean) from household production will be lower in adopter households than in non-adopter households.	Cash crops are commonly criticized for displacing food crops [29].
Access refers to individual’s resources (e.g., income, products for barter) to acquire appropriate foods for a nutritious diet [28].	Household food access will be higher for adopter households than for non-adopter households.	NTX production may increase food access because of higher income [16].
Utilization refers to the fulfillment of physiological needs through nutrition, as influenced by adequate diet, clean water, sanitation and health care [28].	The nutritional status of household members will be higher for adopter households compared to non-adopter households.	It is typically assumed that nutrition improves following NTX adoption due to better economic access [29].
Stability refers to the need for food availability, access, and utilization to be consistent through time despite seasonal cycles or unexpected shocks (e.g., drought) [28]. Here we consider the stability of the local food system as mediated by impacts to agroecosystems.	NTX farming will be associated with declines in regulating services (e.g., biological pest control) posing a risk to food system stability through slow declines in ecological resilience.	Intensive agriculture is recognized as a key driver behind declines in regulating services [30], and NTX production is generally more intensive than the production of corn and bean [3].

### Study location

Guatemala is a challenging and appropriate place to explore the relationship between NTX agriculture and household food security. In Guatemala, poverty affects a staggering 76% of the rural population [31] and 72% of the rural poor engage in farming [32,33]. It is estimated that 24% of the total population in Guatemala is undernourished [34], and 43.4% of all Guatemalan children under the age of five are malnourished [35].

In recent decades, Guatemala’s NTX sector has grown by leaps and bounds [3,36]. Beginning in the early 1980s, the expansion of small-scale NTX production was a central component of U.S. economic assistance policy and was supported through the establishment of favourable trade rules for NTXs and a steady flow of foreign development aid to export-related agencies [5,6,36]. Many smallholders began farming NTX vegetables including snow peas, cauliflower, and broccoli, and later diversified in the 1990s to farm French beans, mini-zucchinis, berries, and other exotic crops. Today, Guatemala is a key player in several NTX markets (e.g., cardamom, winter vegetables, palm oil, fresh fruit) and NTX production is one of the country’s top export earners [24]. Increases in both the quantity and value of NTX exports have also been accompanied by an increase in the land area dedicated to these crops [3,36]. Between 1985 and 2010, the quantity of land dedicated to NTX exports in Guatemala increased by 280 percent [36].

Prior to the introduction of NTXs, agro-export booms in coffee, bananas, sugar, and cattle fundamentally shaped Guatemala’s economic development [37]. However, these agro-export booms tended to exclude smallholders (thus reinforcing Guatemala’s inequitable agrarian and economic structures). In contrast, NTX cultivation in Guatemala has been more inclusive of smallholders [38], as contract farming has helped incorporate small farms into the NTX sector [39]. The production of winter vegetables, in particular, often occurs through contract farming, whereby smallholders enter into contracts with regional representatives of agro-export companies.

**Fig 1:**
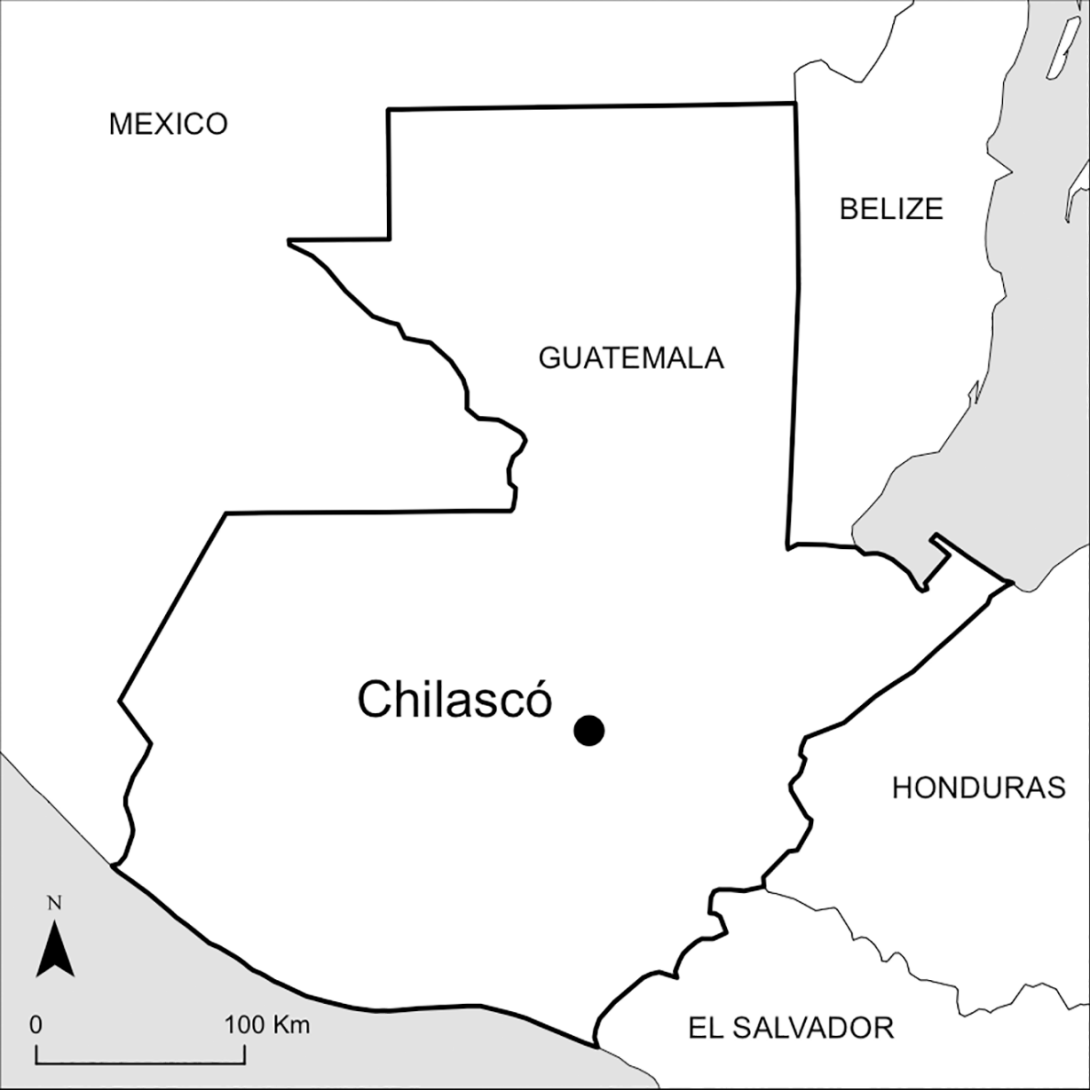
Map showing the location of Chilascó in central Guatemala.

Our research is based in the highland community of Chilascó (15^o^ 07’ 20” N and 90^o^ 06’ 50” W, Fig 1), a hotspot for broccoli production in the central Guatemalan province of Baja Verapaz. Baja Verapaz is characterized by: (i) the central role, both agriculturally and culturally, of corn and beans; (ii) levels of chronic malnutrition in children above the national average (53.3% of children were chronically malnourished in 2008) [35]; (iii) the small size of land holdings–typically under two hectares; and, (iv) the growing importance of smallholder NTX agriculture, particularly broccoli production.

Chilascó is a town of approximately 7,000 people, predominantly ladino, with local livelihoods structured around corn, bean, and broccoli farming. Corn and bean have traditionally made up a majority of the dietary intake of Guatemalans [40] and remain staple crops for the community. Secondary crops grown locally include several varieties of squash, French bean, and to a lesser extent, potato. Smallholder farmers in the community began farming broccoli for international export in the late 1980s, and as of 2011, approximately 75% of local households participated in these schemes. To farm broccoli, farmers enter into written contracts with regional representatives of one of two agro-export companies operating locally. These schemes are open to any local farmers and vary little in their terms. At the start of every three-month broccoli harvest cycle, the companies provide farmers with agricultural inputs (e.g., broccoli seedlings, fertilizers, pesticides) on demand, and farmers are then expected to repay input costs by selling the harvest back to the agro-export company at a pre-determined price.

Chilascó sits within the western buffer zone of the Sierra de las Minas Biosphere Reserve, one of the largest and most ecologically important areas in the country, and lies in a transition zone between mixed conifer and broadleaf cloud forests. At an elevation of 1840 m, Chilascó occupies the highlands of the San Jeronimo watershed and is cool and rainy most of the year. In many ways, the agricultural lands of Chilascó are a farmer’s dream: local soils are deep and historically fertile (rich organic andisols with a sandy loam texture) [41], ample annual rainfall enables a yearlong growing season, and a communal land tenure system helps to ensure that most households have access to small plots of land. However, despite high agricultural activity in the area, both chronic and seasonal food insecurity remain pervasive and longstanding local problems.

## Materials and methods

We used a combination of household surveys, focus groups, and interviews to assess all four dimensions of food security for two types of smallholder (farming less than 2 ha) households: ‘*adopters*’ (households that farm broccoli in addition to corn, bean, and other secondary crops) and ‘*non-adopters’* (households that farm traditional crops like corn, bean, and other secondary crops).

We classified every household in Chilascó as either adopter or non-adopter using a list of households from the year 2010 obtained from the local medical clinic and confirmed by local farmers. A total of 779 households were identified. Of these, 52 households were sampled, including 25 adopter households and 27 non-adopter households. These were selected using stratified random sampling and using ‘adopter’ and ‘non-adopter’ as the stratifying variable. Fieldwork was carried out from May to September 2011.

Study protocols and consenting procedures were approved by the Research Ethics Board in the Faculty of Agricultural and Environmental Sciences at McGill University, and by the Comisión de Estudios Humanos in Guatemala, through coordination with the Center for Studies of Sensory Impairment, Aging, and Metabolism (CeSSIAM). Written informed consent was provided by all research participants, using protocols approved by both Institutional Review Boards. Community participation was approved by the local government of Chilascó.

Two structured surveys were administered to each household in order to collect the data necessary for indicator construction. The first survey was conducted with the female head of household (the person in charge of cooking food) and was designed to collect information on food availability, access, and utilization within the home. The second survey was conducted with the member of the household most familiar with agricultural activities (typically the male head of household) and collected information about agricultural production, income, farming practices, and agricultural trends.

To develop a qualitative context within which to frame survey results [42,43], we also conducted several semi-structured interviews with key informants (e.g., local health and education workers, community leaders, industry representatives. Key informants were selected because of their specific subject matter knowledge and based on recommendations by local contacts. We also held two focus groups to better understand local perceptions about the links between food security and NTX production. An invitation was extended to local women to participate in the first focus group through word of mouth and contacts at the local health center, where we discussed causes and perceptions of local food insecurity. We later held a second focus group with male farmers to discuss agricultural production patterns and perceptions of NTX agriculture.

### Calculating food security indicators

Separate indicators and data collection methods are needed to independently assess each dimension of food security (Table 3). In the sections below, we describe a set of 11 indicators used to assess food availability, access, utilization, and stability. Many of these indicators align with those suggested in a recent review of best practices by Coates (2013) [26].

**Table 3:** The 11 indicators used to assess the food security of households across the four dimensions of food security: Availability, access, utilization, and stability.

Dimension	Indicator	Definition / Explanation
*Availability*	Annual corn and bean production (kg yr^-1^)	Total annual household production of corn and bean.
	Annual corn and bean consumption per adult equivalent (kg yr^-1^ per adult equivalent)	The amount of corn and bean cooked (kg) in the household for a ‘good’ week and a ‘bad’ week (kg), taken as an average, multiplied by 52, and then divided by the number of household adult equivalents Calculated as [((kg_bad_ +kg_good_)/2)*52] / (household adult equivalents).
*Access*	Annual net household income (USD yr^-1^)	The sum of annual agricultural sales, the imputed value of staple crop consumption, off-farm wage income and miscellaneous income sources (e.g. sale of wild edible plants, small livestock) minus agricultural input costs (pesticides, fertilizers, hired labour, seeds, and technology).
	Household Food Insecurity Access Scale (HFIAS)	Used to classify households as either food secure or mildly, moderately, or extremely food insecure [44]. It is based on a respondent’s subjective experience of household food insecurity over the previous 30 days. Results may also be displayed as a continuous score (range: 0 (food secure) to 27 (extreme food insecurity)).
	Household Hunger Scale (HHS)	Used to classify households as experiencing three levels of hunger (little to no, moderate, or severe) [44]. It is based on a respondent’s subjective experience of hunger over the previous 30 days.
	Months of Adequate Home Food Provisioning (MAHFP)	The number of months in a given year that a household self-reports adequate access to food for consumption (either through household production, purchase, or aid) [45].
	Household Dietary Diversity Score (HDDS)	The number of distinct food groups (up to 12) that were eaten within the household the previous day [46].
*Utilization*	Food Consumption Score	A composite score based on the dietary diversity, food frequency, and weighted nutritional importance of different food groups [47]. The FCS was calculated for one-child (randomly selected) between the ages of 1 and 8 years old per household.
*Stability*	Environmental Impact Quotient (EIQ) for pesticide use (EIQ ha^-1^)	Employed here as a proxy for the ecosystem service of biological pest control, the EIQ is a continuous measure of the environmental impact of pesticide use per hectare based on the type, toxicity, and application rate of pesticides in a year [48].
	Nitrogen application (kg N ha^-1^ yr^-1^) Phosphorus application (kg P ha^-1^ yr^-1^)	Employed here as a proxy for the ecosystem service of nutrient cycling, and defined as the kilograms of nitrogen (N) and phosphorus (P) applied per hectare over a 12 month period. The kilograms of N and P were calculated using the known Nitrogen: Phosphorus: Potassium (N:P:K) ratios for each fertilizer type applied by households over the previous 12 months.

#### Food availability

We employed two indicators to measure food availability: 1) total annual household production of corn and bean (kg yr^-1^); and 2) total annual corn and bean consumption per capita (kg yr^-1^ per capita). The annual production of both corn and bean (kg yr^-1^) by each household was calculated by asking farmers about harvest quantities over the previous 12 months. Farmers were also asked about the harvested quantities of other crops (e.g., broccoli, potato), however, because these crops are primarily sold for monetary gain, we consider their contribution to food security within the section on food access through income.

Staple food production is only one aspect of food availability, and so additional measures are needed to understand the food actually available to any given household [49]. We estimated the annual corn and bean consumption per adult equivalent in each household (kg yr^-1^ per adult equivalent) using a proxy based on the weekly consumption of staples [15]. We first asked participants to provide estimates of the amount of corn and bean cooked within the household for a ‘good’ week and a ‘bad’ week. These values were then averaged to find the amount cooked during an average week, multiplied by 52 weeks, and then adjusted to reflect annual household consumption per adult equivalent using standard methods [40]. This approach provides a rough indication of the corn and bean cooked within each home and available to each individual annually, regardless of the food source.

#### Food access

We employed five indicators of food access: 1) annual net household income (USD yr^-1^); 2) the Household Food Insecurity Access Scale (HFIAS); 3) the Household Hunger Scale (HHS); 4) the Months of Adequate Home Food Provisioning (MAHFP); and 5) the Household Dietary Diversity Score (HDDS). All five indicators were calculated on the basis of survey questions.

Income is a common proxy used to measure food access. We calculated annual net household income as the sum of agricultural and non-agricultural wages, annual agricultural sales, the imputed value of staple crop consumption, and miscellaneous income sources (e.g., sale of wild edible plants, foreign remittances) minus agricultural input costs (pesticides, fertilizers, labour, seeds, technology, land) [50]. The monetary value of crop consumption was imputed using average sale prices in the region [51]. We also calculated net on-farm income as total annual agricultural production multiplied by the average price of products, minus the cost of inputs (pesticides, fertilizers, seed, and technology) and the cost of labour. This calculation excluded imputed labour.

Income is a food security determinant; it is not a food security outcome. A robust analysis of the food access dimension also requires indicators of food security outcomes. For this reason, we calculated the Household Food Insecurity Access Scale (HFIAS), based on a set of nine questions designed to provide a single measure of a household’s ability to access food [52]. Following the methodology of Coates et al. (2007), we classified households into four categories of food insecurity based on their continuous HFIAS scores: food secure, and mildly, moderately, and severely food insecure [52].

We also assessed food access using an indicator called the Household Hunger Scale (HHS). The HHS is a derivative of the HFIAS used to specifically assess hunger (itself only one expression of food insecurity). Following the methodology of Deitchler et al. (2011), we classified households as experiencing little to no hunger, moderate hunger, or severe hunger [44].

We also assessed the desired outcome of improved food access–improved household food consumption–using two indicators called the Months of Adequate Home Food Provisioning (MAHFP) and the Household Dietary Diversity Score (HDDS), respectively. The MAHFP is measured as the number of months over the previous 12 months that a household self-reports having had adequate access to food for consumption (through household production, purchase, or aid) [45]. The HDDS is an indicator of household-level dietary diversity that has been validated as a meaningful measure of household food access: households consuming a more diverse diet (as assessed by the HDDS) were shown to have greater access to food, as indicated by food consumption and expenditure data [53]. To calculate the HDDS, we asked the female head of household whether or not a specific list of foods had been prepared and eaten in the household the previous day. We then tallied the number of distinct food groups (up to 12) that had been eaten within the household the previous day [46].

#### Food utilization

Adequate household access to food does not necessarily correspond to adequate food utilization and nutritional outcomes at the individual level. To measure the *utilization* dimension, we used a single proxy of the dietary quality of children called the Food Consumption Score (FCS) [47]. The FCS is a composite score based on dietary diversity, food frequency, and the weighted nutritional importance of different food groups [47], and is calculated on the basis of standardized survey questions. Data on dietary diversity and food frequency have proven to be reliable proxy indicators of diet quality across a range of settings [54,55].

We calculated the FCS for one child (randomly selected) between the ages of 1 and 8 years old per household, excepting households with no children in the age cohort. To calculate the FCS, we asked mothers about the type and frequency of foods eaten by a child the previous day [47].

#### Food stability

Although the dimension of “stability” has a cross-cutting influence on food availability, access, and utilization [56], this dimension is often underrepresented or ignored in food security analyses [19,20,57], in part because of a lack of clear, agreed-upon indicators [49]. Given a lack of generally agreed-upon measures, for the purposes of this paper, we propose using the ecosystem services framework as an entry-point to better understanding and measuring food system stability.

We employ indicators of ecosystem services–defined as the benefits that humans derive from nature [30]–in order to explore how agroecosystems and their specific services can contribute to food security in the long-term. It has been shown that the ecosystem services generated by agroecosystems, such as biological pest control and nutrient cycling, are a key part of how agroecosystems can enable long-term food security [30,58,59]. Recognizing that the best indicators of stability are often slowly changing ecological variables such as regulating ecosystem services [58], we assessed two slowly changing regulating ecosystem services–biological pest control and soil nutrient regulation–to better understand food system stability.

To investigate the status of biological pest control in the parcels of adopter and non-adopter households, we employed an indicator called the Environmental Impact Quotient (EIQ) of pesticide use [60]. The EIQ is widely used to estimate environmental hazards associated with agricultural pesticide use [48]. It is a continuous measure of the environmental impact of pesticide use per hectare and a composite hazard indicator that includes dimensions of ecological, farmworker, and consumer exposure risk to pesticides used in crop production [60,61]. The higher the EIQ score per hectare, the higher the hazard posed to the social-ecological system. We hypothesize that higher EIQ scores indicate lower provision of the service of biological pest control, as natural pest enemies are often eliminated through increasing pesticide use [62]. We calculated the average EIQ score per hectare for each household in the sample, based on survey questions detailing seasonal pesticide use using the methodology of Kovach et al. 1992. The EIQ score per ha was calculated as a function of pesticide dosage, application rate, and a standard environmental impact value assigned to each active ingredient [60].

As a proxy for the ecosystem service of soil nutrient regulation, we also asked farmers about the types and quantities (kg) of inorganic and organic fertilizers applied to their fields over the previous 12 months and then calculated the total nitrogen (kg N yr ^-1^) and phosphorus (kg P yr ^-1^) applied to a household’s agricultural land based on N:P:K ratios. This indicator is only an entry point to understanding soil nutrient regulation, as fertilizer use may indicate as much or more about farmer responses to perceived soil nutrient levels than it does about soil nutrients or nutrient regulation.

### Statistical analyses

To determine whether or not there are significant differences in the food security status of adopters and non-adopters for each of the four dimensions, we tested for significant differences between these two groups for each of the different indicators of each dimension. We conducted all statistical comparisons using SPSS Statistics Version 17.0 (SPSS Inc., Chicago, IL) and present the level of significance at p < 0.1 and p < 0.05. Data are reported as mean ± SEM (the standard error of the mean) unless otherwise stated. We compared continuous data using Student’s *t*-tests and two-way analysis of variance for normally distributed data, and used the non-parametric Mann-Whitney U test for non-normally distributed data. In the case of multiple comparisons with a significant test result, we conducted post-hoc Tukey tests. We used chi-squared tests to compare nominal or ordinal level data. We used Phi to measure the strength of association for cross-tabulations of nominal variables with only two categories, whereas we used Cramer’s V for *nominal* by *nominal* or *ordinal* by *nominal* cross-tabulations with more than three categories per variable.

## Results

The general characteristics of households–including household size, agricultural land holdings, the average number of children per household, and the ages and literacy levels of female and male heads of household–were not significantly different between adopter and non-adopter households (Table 4). Access to education in Chilascó is limited; 69% of female heads of household, and 42% of male heads of household, were illiterate. The primary occupation of male household heads in adopter households was household agriculture, whereas significantly more non-adopters relied on local agricultural wage labour as the primary occupation of male household heads (Chi-square, *X*^2^ (3, N = 52) = 7.23, p = 0.065, V = 0.380).

**Table 4:** Summary of household characteristics for non-adopter (N = 27) and adopter (N = 25) households.

	Non-adopter		Adopter		p
	Mean	SEM	Mean	SEM	
Household size (# of members)	5.89	0.43	6.72	0.45	0.183
• Household size (# of members, adjusted)	4.05	0.23	4.18	0.26	0.724
Agricultural land (ha)	0.75	0.14	1.06	0.13	0.103
Children (#)	2.67	0.33	3.24	0.35	0.240
*Male household head*					
• Age (years)	45.4	2.7	41.4	2.7	0.309
• Primary occupation is household agriculture (%)	56		88		0.065*
• Primary occupation is hired agricultural labour (%)	36		8		
• Illiteracy (%)	52		32		0.148
*Female household head*					
• Age (years)	41.4	2.3	39.1	2.5	0.498
• Primary occupation is domestic work (%)	85		88		0.566
• Illiteracy (%)	74		64		0.432

Statistically significant

* P <0.1

Our results indicate that non-adopters had 0.75 ± 0.14 hectares of agricultural land and adopters had 1.06 ± 0.13 hectares, including both rented and owned land. According to a classification of family farms in Guatemala done by Fradejas and Gauster (2006), these households would be classified as “infra-subsistence” (<0.7 ha) or “subsistence” (0.7–7 ha) [63]. Assuming that approximately 1.17 hectares (11 739 m^2^) are required to support a subsistence agricultural lifestyle for an average-sized family in Guatemala [25,64], the majority of households in our sample had insufficient land holdings to fully maintain a subsistence agricultural lifestyle.

### Differences in food availability between NTX adopters and non-adopters

Food availability, as measured by staple food production and staple food consumption, did not differ significantly between groups. There were no statistically significant differences in the amounts of corn (p = 0.838) or bean (p = 0.282) grown annually by adopter and non-adopter households (Table 5). Corn and bean yields obtained by both groups were consistent with the national average yields for both crops [65]. The average broccoli yield obtained by adopter households was also consistent with the national average (Table 5) [65]. There were no statistically significant differences in annual corn (p = 0.705) or bean (p = 0.993) consumption per adult equivalent between groups (Table 5).

**Table 5:** Results for indicators of food availability, access, and utilization for non-adopter (N = 27) and adopter households (N = 25) in Chilascó, Baja Verapaz, Guatemala.

	Non-adopter		Adopter		p
	Mean	SEM	Mean	SEM	
**AVAILABILITY**					
*Total production (kg yr*^*-1*^*)*					
• Corn	761	265	824	148	0.838
• Bean	180	58	195	42	0.282
• Broccoli	-	-	10917	2073	-
*Yield (kg ha*^*-1*^*)*					
• Corn ^a^	911	110	1156	162	0.322
• Bean ^a^	266	40	287	44	0.517
• Broccoli	-	-	9666	1321	-
*Annual consumption of staples (kg yr*^*-1*^ *per adult equivalent)*					
• Corn	219	25	231	22	0.705
• Bean	46	9	36	4	0.993
**ACCESS**					
*Household income (USD yr*^*-1*^*)*					
• Net annual income	2100	257	3133*	489	0.062*
• Net on-farm income	382	137	1572**	423	0.011**
• Off-farm income	1717	198	1560	273	0.641
*Household Food Insecurity Access Scale*					
• HFIAS (continuous)	9.48	1.10	8.64	1.16	0.601
• HFIAS (categorical, mode)	Moderate food insecurity		Moderate food insecurity		0.721
*Household Hunger Scale (categorical*, *mode)*	Little hunger		Little hunger		0.810
*Months of Adequate Home Food Provisioning*	7.48	0.56	7.72	0.68	0.786
*Household Dietary Diversity Score*	7.59	0.37	7.40	0.41	0.726
**UTILIZATION**					
*Food Consumption Score* ^a^	62.8	4.1	65.6	4.4	0.651

Statistically significant

* P <0.1

** P <0.05

^a^ Food consumption score calculated for reduced sample size of non-adopter (N = 19) and adopter (N = 20) households with at least one child in the age range (1–8 years).

### Differences in food access between NTX adopters and non-adopters

Only one of the five indicators of food access–net annual household income–differed significantly between groups, as adopter households had significantly higher net annual incomes (Independent Samples t-test; t (50) = -1.91, p = 0.062) (Table 5). On average, there was a 40% difference in net annual income between groups. Adopter households also earned significantly more on-farm income compared to non-adopters (Mann-Whitney U = 199.00; z = 2.54; p = 0.011), however off-farm income did not differ significantly between groups (p = 0.641).

The average daily per capita incomes of non-adopters (1.05 ± 0.13 USD per capita day^-1^) and adopters (1.52 ± 0.30 USD per capita day^-1^) in Chilascó were below the absolute and general poverty lines established for Guatemala and internationally in 2011. Using an exchange rate of 1 USD = 7.823 Guatemalan Quetzales (GTQ)–as of 2011-09-09, accessed from xe.com–the mean daily per capita income of non-adopters and adopters was 5.06 ±0.80 GTQ per capita day^-1^and 8.99 ±1.95 GTQ per capita day^-1^, respectively. The Guatemalan general rural poverty line was 18.78 GTQ day^-1^ per capita in 2011. The international general poverty line (($2.00 day^-1^ per capita, 2005 Purchasing Power Parity) [66] corresponds to GTQ 13.01 day^-1^ per capita, using the exchange rate above and the methodology described by Sillers (2006) [67].

The HFIAS shows that the experience of household food insecurity did not differ significantly between groups, when treated as either a continuous (p = 0.601) or as a categorical variable (p = 0.721). According to this indicator, 8% of households in the sample were food secure, 13% were mildly food insecure, 44% were moderately food insecure, and 35% were extremely food insecure. Looking across the nine questions comprising the HFIAS module (Table 6), there was only one significant difference in the response rate for a question between groups. Non-adopter households had a significantly higher rate of responding “yes” to the question of whether or not they had to eat food they did not want to out of necessity (Chi-square, *X*^2^ (1, N = 52) = 7.63, p = 0.01, V = 0.39). In both groups, anxiety about food was the most common expression of food insecurity (experienced by 89% of households), followed by insufficient dietary quality (70%), and lastly by the experience of insufficient food intake and associated physical consequences (33%) (Table 6). Looking at more extreme manifestations of food insecurity, 83% of households experienced ‘little to no hunger’ and 17% of households experienced ‘moderate’ hunger, according to the Household Hunger Scale, itself a derivative of the HFIAS. There were no cases of ‘severe’ hunger, according to this indicator, and no significant differences in the experience of hunger across groups (p = 0.810).

**Table 6:** Itemized responses to the Household Food Insecurity Access Scale (HFIAS), for non-adopter (N = 27) and adopter (N = 25) households in Chilascó, Baja Verapaz, Guatemala.

	Question	Domain	Non-adopters (%)	Adopters (%)
1.	Worry about food quantity	Anxiety about food supply	89	88
2.	Unable to eat preferred foods	Insufficient quality	78	80
3.	Eat just a few kinds of food	Insufficient quality	81	68
4.	Eat foods that you do not want to eat	Insufficient quality	74	36**
5.	Eat less than necessary during meals	Insufficient food intake	67	64
6.	Eat fewer meals per day	Insufficient food intake	41	44
7.	No food to eat in home	Insufficient food intake	26	16
8.	Go to sleep hungry	Insufficient food intake	30	20
9.	Full day without food	Insufficient food intake	15	8

The percentage of households that responded “yes” to each specific occurrence question from the scale are listed, as related to three domains of food insecurity: 1) anxiety and uncertainty, 2) insufficient diet quality, and 3) insufficient food intake. Statistically significant

* P <0.1

** P <0.05.

According to another indicator of food access, the Months of Adequate Home Food Provisioning, both adopter and non-adopter households reported that they had inadequate food (both produced and purchased) over a least four months of the previous year. There was no significant difference in the reported number of months of adequate home food provisioning between groups (p = 0.786 Table 5). June and July are the ‘hunger months’ in Chilascó, and even adopter households with higher annual incomes struggled to feed household members adequately.

There was no significant difference in the mean household dietary diversity scores for non-adopter and adopter households (p = 0.726; Table 5). Local diets are based on the consumption of staple cereals, legumes, sugar and honey, and coffee. Overall, households in the lowest dietary diversity tercile (based on HHDS scores) ate a restricted diet of cereals, legumes, and sugar/honey. The food items that make up the additional diversity of higher scoring diets include oils, vegetables, fruits, and eggs. Households with the highest dietary diversity also ate meats and roots or tubers (typically potatoes). According to a two-way analysis of variance, there was a significant main effect of income on the household dietary diversity score (F_2,46_ = 5.71, p = 0.006), with households in the top income tercile having significantly higher dietary diversity compared to households in the bottom tercile (Tukey HSD, p = 0.009). There was no significant main effect of household type (adopter versus non-adopter, F_1, 46_ = 1.41, p = 0.41) and no interaction effect (F_2, 46_ = 0.09, p = 0.920).

### Differences in food utilization between NTX adopters and non-adopters

The food consumption scores of children living in non-adopter and adopter households were not significantly different (p = 0.651) (Table 5), based on a random sample of 39 children between the ages of 1 and 8. According to international benchmarks [55,68], 95% of these children had ‘acceptable’ food consumption scores (scores over 42) with only two reported cases of ‘borderline unacceptable’ (scores of 28–42) and ‘poor’ (scores of 0–28) food consumption scores. There was a significant main effect of total annual income on the food consumption score of children (F_2, 33_ = 3.18, p = 0.055). Children living in households in the highest income tercile had significantly higher food consumption scores compared to children living in lower income households (Tukey HSD, p = 0.042). However, there was no significant main effect of household type (adopter; non-adopter) (F_1,33_ = 0.01, p = 0.918) and no interaction between these two factors (F_2,33_ = 0.07, p = 0.933).

### Differences in food system stability between NTX adopters and non-adopters

#### Biological control of pests

While all adopters (N = 25) applied pesticides in the previous 12 months, only 56% (N = 15) of non-adopters used pesticides. Adopters had a significantly higher environmental impact quotient (EIQ) per hectare associated with pesticide use compared to non-adopters (Mann-Whitney U = 146.000; z = -3.097, p = 0.002). The median EIQ per hectare for non-adopters was 4 whereas for adopters it was nearly 52 (Table 7).

**Table 7:** Results for indicators of food stability for non-adopter (N = 27) and adopter households (N = 25) in Chilascó, Baja Verapaz, Guatemala.

	Non-adopter			Adopter			p
	Median	Mean	SEM	Median	Mean	SEM	
*Biological control of pests*							
• Environmental Impact Quotient (EIQ ha^-1^)	4.23	104.7	52.4	51.6	128.6	44.4	0.002**
*Nutrient cycling*							
• N application (kg ha^-1^ yr^-1^)		116	34		221	25	0.018**
• P application (kg ha^-1^ yr^-1^)		28	8		56	6	0.009**

Statistically significant

* P <0.1

** P <0.05 using t-test of differences in mean values.

According to survey results, farmers were overwhelmingly of the opinion that there were more pests in Chilascó in 2011 than 5 years before; adopter households (96%) were more in agreement with this yes/no statement than non-adopters (81%) (Chi-square, *X*^2^ (1, N = 52) = 5.069, p = 0.079, V = 0.315). Farmers tended to attribute this to, in order of decreasing frequency: 1) increasing intensity and expansion of broccoli production (n = 9); 2) increasing pesticide resistance by pests (n = 8); 3) leftover crop residue from broccoli and cabbage production (n = 5), and 3) poor soil fertility in general (n = 3). This follow-up question was not posed to every farmer, which accounts for response levels below the sample size. Local farmers identified clubroot, a disease affecting Brassica crops and caused by the parasite *Plasmodiophora brassicae* Woronin, as being their chief agricultural concern during a focus group. The presence of clubroot was reported by approximately 60% of adopter households and by 22% of the non-adopter households who rent out their lands for broccoli farming.

#### Nutrient cycling

On a per-hectare basis, adopters applied significantly more chicken manure (Independent samples t-test, t (50) = -2.554, p = 0.014;Table 7) and close to three times more inorganic fertilizer (Independent samples t-test, t (50) = -2.577, p = 0.013) than non-adopters. Adopters applied significantly more nitrogen per hectare than non-adopters (Independent samples t-test, t (49) = -2.45, p = 0.018). The phosphorus application rate was also significantly higher–approximately double–for adopters compared to non-adopters (Independent samples t-test, t (49) = -2.725, p = 0.009).

### Qualitative results

Qualitative data from key informant interviews and focus groups indicated that multiple variables influence food security outcomes in Chilascó, including poor health, low education levels, demanding agricultural labour loads, and environmental exposure to the wind and rain due to poor housing. When asked why malnutrition and hunger persist in Chilascó despite the apparent productivity of local farmlands, community members stressed three key issues: i) the production of vegetables for export rather than for household consumption; ii) parental neglect of children while farming and poor hygiene; and, iii) insufficient education, especially for mothers. Key informant interviews described access to fresh, nutritious food as a key challenge in the community: “In Chilascó, we are blessed to have many types of vegetables but many times our people do not consume them. They prefer to give them to the market.” Another interviewee described how adopters “plant broccoli and can then earn money, but they do not necessarily buy good food with the profit”. Multiple study participants expressed concern that male household heads frequently purchased non-essential items, particularly alcohol, in lieu of nutritious foods. In the words of one interviewee, “I don’t have the money…it costs…the food and the vices…it all costs”. Poor hygiene and inadequate sanitation were cited as factors influencing food utilization. One woman described how food insecurity is linked to “the poor handling of everything” and how, poor hygiene and sanitation is then linked to diarrhea (the most common sickness treated in the local clinic). Focus group participants reported that that improper handling of chicken manure used to support broccoli production fosters housefly populations, contaminates local food, and contributes to diarrhea and malnutrition. Locals report that houseflies were not a major problem prior to broccoli production. A focus group with male farmers also revealed concerns related to the productivity of local agricultural land, owing to perceived soil degradation over time. Overall, participants found it difficult to generalize differences in the food security status of non-adopter and adopter households, and instead emphasized the importance of household-level allocation decisions, as well as education and general health as key determinants of food security status.

## Discussion

In our study, the food security of NTX adopters mostly did not differ from that of non-adopters, except for the dimension of food access, which differed due to increased income for NTX adopters. While adopters earned significantly more income (40%) than non-adopters, there were no significant differences in other measures of food availability, food access, or food utilization for adopters relative to non-adopters. Adopters used significantly more agrochemicals than non-adopters, which may be associated with declines in the regulating ecosystem services of biological pest control and nutrient cycling over the long-term.

### Food availability

Broccoli farming did not reduce the staple food production of adopter households relative to non-adopters. Although Webb et al. (2016) found that farming NTX crops reduced the amount of land dedicated to growing staple crops in another rural Guatemalan community [25], we found no evidence that broccoli production crowds out the annual *production* of corn and bean by adopter households in Chilascó. Given that there was no significant difference in the average size of land holdings between groups, staple crop production by adopters is likely maintained by multiple factors, including: (i) a shift by adopters towards planting corn that can be harvested within four months (instead of a traditional nine-month variety), allowing them to grow both broccoli and corn on the same land in any given year; (ii) the preference of non-adopters to continue farming nine-month corn in order to maintain low farm input costs and because of taste; and, (iii) a possible spillover effect of broccoli fertilization on the yields of staple crops [17]. In our study design, we had initially hypothesized that corn and bean production would be displaced by broccoli production, however, our results are instead consistent with studies which found that the household production of staple crops can be maintained or even increase with NTX adoption [16,22,23].

### Food access and utilization

In Chilascó, the average net annual income of adopters was 40% higher than that of non-adopters. This finding supports our initial hypothesis, reflects previous evidence that NTX adoption is associated with higher household incomes in countries including Guatemala [16,17,69,70], Gambia [71], Kenya [72], the Philippines [73], and India [74], and generally supports the rationale behind initiatives like USAID’s Feed The Future that advocate for NTX agriculture as a means to improve income and thus food access [70].

Nevertheless, our results indicate that higher incomes associated with NTX agriculture did not coincide with improvements in other indicators of food access and food utilization. The majority of adopters and non-adopters were categorized as moderately to extremely food insecure according to the HFIAS, and, on average, households reported being without adequate food for four months of the year. Households felt anxious about the food supply and also coped with food of insufficient quality and the physical consequences of these deficits–regardless of whether or not they chose to partake in NTX agriculture.

In key informant interviews, regional agricultural and food security experts characterized Chilascó as a puzzle, and wondered how food insecurity persists despite significant local agricultural production and land ownership. Webb et al. (2016) propose that other NTX-farming communities in Guatemala facing this apparent paradox may be likened to food deserts [25]; contexts with “relatively poor access to healthy and affordable food” [75]. Our qualitative assessment of food security dynamics in Chilascó supports this proposition, whereby household access to sufficient, nutritious food is impeded by the export of vegetables outside of the community as well as intra-household factors including purchasing decisions. Notably, we found that male household heads often used NTX profits to purchase non-essential items or alcohol in lieu of additional food. This supports the work of Katz (1995), who argues that male-biased NTX market structures threaten to deepen asymmetrical intra-household resource distribution and thus limit improvements in food access or nutrition [69].

Interestingly, the Food Consumption Scores of children suggest that the dietary quality of children was mostly adequate across groups, regardless of adoption status. Looked at one way, we might conclude that although adopters were not better off than non-adopters as hypothesized, it is a positive sign that children’s food consumption scores were largely considered adequate in both groups. However, our understanding of nutritional outcomes using this indicator is limited because current benchmarks for the FCS may significantly underreport cases of inadequate food consumption in Guatemala [47], and because the FCS gives only a snapshot of food consumption over the previous week for one individual. Importantly, our qualitative findings indicate that food utilization in Chilascó was often negatively affected by poor hygiene, inadequate sanitation, and the use of organic manures for broccoli production.

That higher income–a food security determinant commonly recognized as an indicator of food access–is not associated with better food access or food utilization implies that policies that aim to improve income alone will not be adequate for improving food security outcomes. Our results are consistent with an important study by von Braun et al. (1989) [16], who found that NTX adoption in the Central Highlands of Guatemala significantly increased household income and yet had no visible positive effects on nutrition. Other studies have also found that NTX agriculture does not necessarily lead to improved dietary energy and protein intake [17] or improved nutritional outcomes in Guatemala [25,76].

Despite the fact that positive relationships between income, food access, and food utilization cannot be assumed, it is also true that sometimes income gains from NTX farming *do* lead to improvements in food security [29,77]. Schuftan (1998) argues that although multiple studies suggest that household income alone cannot lead to improved food security and nutritional status, income does have an important role to play in improving food security, however typically for the lowest income decile households or for the already extremely malnourished [77].

### Food stability: A long-term perspective

Our results suggest that broccoli production may be undermining the ability of local agricultural systems to naturally control pests and regulate nutrients, recognizing that adopters used significantly higher quantities of pesticides and fertilizers than non-adopters. In Chilascó, escalating pesticide use and mounting pest problems indicate that biological pest control is being threatened by agricultural intensification. The higher environmental impact quotient (EIQ) of pesticide use associated with adopters suggests that the NTX model is driving these changes. Of the adopters surveyed in Chilascó, 96% reported higher pest levels between 2006 and 2011, a trend that evokes an image of the “pesticide treadmill” in which increasing pesticide use leads to limited natural biological control, leading to more pesticide use [78,79]. This pesticide treadmill has been found to affect other NTX producers in Guatemala [80] and Central America [81].

The degradation of agricultural ecosystem services that this implies has consequences for food security because undermining natural assets can limit the capacity of households to generate future-income and avoid social vulnerability [82]. The agronomic problems confronting Chilascó are similar to those in other areas with intensive NTX production throughout Guatemala, and include dramatic increases in pest problems and pesticide resistance [80,83]; declines in soil quality [83], and the toxicological contamination of crops [4]. Working in a region of Guatemala where farmers grow snowpeas (an NTX crop), Carletto et al. (2011) found that the prolonged and excessive use of fertilizers and pesticides contributed to soil degradation and, ultimately, a 30% decline in snow pea productivity between 1985 and 2005, forcing some snowpea farmers to abandon production [3]. A key question is thus to what extent these trends may impact the long-term viability of broccoli production in Chilascó and the food security of households farming NTX crops over time.

### Study limitations

There are some limitations related to our choice of study design. In particular, the relatively small sample size (52 households) makes the generalization of findings to reflect the subgroups in the population difficult [84]. Nevertheless, the results of our case study may provide constructive insights into food security and how we measure it [43], and adds to a growing body of research linking NTX agriculture with food security outcomes in Guatemala. In this case, while farmers in Chilascó may be only partially representative of farmers in other communities (in being poor, isolated, and generally food insecure), the message of our study, that food security must be evaluated systematically across all four dimensions, should be one of great concern to policy-makers around the world who are considering promoting NTX agriculture to address food insecurity.

Another limitation relates to the choice of the ‘household’ (undifferentiated) as the unit of study, as this ignores intra-household factors and questions of gender, age, and power. There is therefore a possibility that households were classified as food secure, when individual members were not, or vice versa [85]. Finally, the cross-sectional design of this study–which classifies the population of Chilascó into a dichotomy of adopters and non-adopters–ignores how the timing and duration of NTX adoption may influence household welfare levels or whether adopter and non-adopter families are interacting in ways that might affect their food security (e.g., if NTX farming provides agricultural labour jobs that help increase food security for both groups) [3].

Taken together, the limitations described do not invalidate the conclusions of the study but rather call for more diversified information in the future.

### Recommendations for future research and policy-making

Selecting appropriate (and adequate) indicators for the dimensions of food security remains a stubborn challenge. Indeed, the international community “has not found a way to identify how, when, and where different facets of the [food security] concept are more important than others” [27]. More work is needed to identify indicators that reflect different dimensions and to develop a holistic, systematic approach to measurement [26]. The question of how to measure the dimension of stability stands out as a particularly important problem area. While indicators of ecosystem services–particularly regulating services–offer a promising new avenue to this end, a concerted effort to develop social, economic, and environmental indicators of stability is needed. Our vision for food security measurement is an approach that examines multiple dimensions, considers a full suite of social, economic, and ecological indicators, and follows both short- and long-term trends. As more systematic approaches to food security assessment are implemented, we hope that lessons learned will help the international community to thoroughly investigate the strengths and limitations of the NTX model in terms of alleviating household food insecurity. With this in mind–and recognizing that positive relationships between NTX adoption, income, food security, and nutrition cannot be assumed–further empirical research is needed, particularly long-term studies that address both the timing and duration of NTX adoption. Longitudinal studies with panel data collection are needed to estimate the causal impacts of NTX adoption on food security outcomes.

Meanwhile, policy-makers continue to promote NTX agriculture in Guatemala and across poor, food insecure parts of the world [70]. These agencies would do well to consider–and measure–which aspects of food security change with NTX adoption, which do not, and the timeframe of any changes. In particular, if NTX adopters have higher incomes but similar levels of other indicators of food security, we might ask ourselves whether NTX adoption is having the results we envision for local communities. Overall, more holistic approaches to food security assessment may help to better identify potential food security trade-offs and improve the targeting of interventions, while also shedding light on the benefits and drawbacks of NTX agriculture as a strategy to alleviate smallholder food insecurity in developing nations.

## Conclusions

Sweeping arguments are often made either for or against the potential for NTX agriculture to improve the food security of smallholder farmers in developing countries. However, the results of our study–the first to systematically compare the food security status of adopters and non-adopters across four dimensions of food security–indicate that these arguments may be lacking in necessary nuance. Our results show that the food security status of adopters and non-adopters in Chilascó varies depending on the dimension of food security considered. It is therefore critically important to consider how NTX production may affect all four dimensions of food security, while recognizing that different indicators paint different pictures of household food security. Our research moves beyond a dualistic understanding of food security outcomes (better/worse) toward an analytical framework that considers food security within a matrix of interactions and potential trade-offs. As the commercialization of smallholder agriculture expands across Guatemala, understanding these interactions has important implications for the food security and wellbeing of the rural poor.

## Supporting information

S1 File Household survey questions. (DOCX)

S2 File Food security data. (XLSX)
