# Supplementary material for: Reconsidering non-traditional export agriculture and household food security: A case study in rural Guatemala
Source: PLoS One. 2018 May 24;13(5):e0198113. doi: 10.1371/journal.pone.0198113 (PMC5967828; doi:10.1371/journal.pone.0198113)
Supplement: S1 File — (DOCX) [file pone.0198113.s001.docx]

**SUPPLEMENTARY INFORMATION: HOUSEHOLD SURVEYS**

This document provides the original household survey questions in both english and spanish. Two surveys were administered with each household: A) Food security survey, and B) Agriculture Survey.

***FOOD SECURITY SURVEY***

***I. GENERAL HOUSEHOLD INFORMATION***

**HOUSEHOLD COMPOSITION AND EDUCATION**

| **1**. Name of the household head who eates and sleeps regularly in this household |  | | |
| --- | --- | --- | --- |
| **2**. Please specify the names, ages, and sexes of all people who eat and sleep regularly in this household. |  |  |  |
| **3**. What level of studies have you completed (...)?  1) None 2) Primary (Grade 1 -3) 3) Primary (Grade 4- 6) 4) Basico 5) Diversificado –incomplete 6) Diversificado – complete 7) Superior – incomplete 8) Superior - complete |  | | |
| **4**. Can you read and write? 1) Yes 2) A Little 3) No |  | | |
| **5**. Have you worked in agricultural parcels for the benefit of the household in the past 12 months? 0) No 1) Yes |  | | |

**HOUSEHOLD AND LAND TENURE**

| **6.** The home where you live is (…): 1) Owned by the household and completely paid off 2) Owned by the household but with regular payments🡪 1B 3) Inherited or gifted 4) Loaned or borrowed 5)Rented 🡪 1B 6)Other |  |
| --- | --- |
| **7**. How much do you pay per month in rent? (quetzales) |  |
| **8**. How long have you lived in the community? (year of the arival) |  |
| **9**. Is there someone from this household who works agricultural lands for household production? 0) No 1) Yes |  |
| **10**. Who is the most informed person about agricultural activities on the part of the household? (name) |  |
| **11.** Are you the person most aware of household agricultural activities? 0) No 1) Yes |  |
| **12.** How much agricultural land does the household currently possess or rent? (manzanas) |  |
| **13**. The terrain where you farm is (…): Please specify from largest to smallest land area 1)Your own 2)Rent 3)Communal 4)Private 5)The state’s 6)Municipal |  |
| **14**. What type of document credits the possession of this land? 1)Receipt 2)Public deed 3)Registered deed 4)Municipal certificate 5)Communal property title 6)Other, specify? 7)No document |  |

**HOUSEHOLD MATERIALS, WATER, AND ENERGY**

| **15**. What are the exterior walls of the house primarily made of? 1)Blocks 2)Concrete 3)Wood 4)Bajareque 5)Metal lamina 6)Other, what? | |  | | | |
| --- | --- | --- | --- | --- | --- |
| **16**. What material is the roof primarily made of? 1)Metal lamina 2)Tiles 3)Cement 4)Palms or plants 5)Other, what? | |  | | | |
| **17.** What material is the interior floor made of? 1)Cement or mud brick 2)Cement 3)Bare ground, dirt 4)Ceramic 5)Wood 6)Other, what? | |  | | | |
| **18**. What type of stove do you have? 1)Improved stove 2)Ground fire 3)Polleton 4)Propane stove 5)Kerosene stove 6)Other, what? | |  | | | |
| **19**. What type of sanitation system do you have? 1)Toilet connected to drainage network 2)Latrine or open well 3)Washable toilet 4)None 5)Other, what? | |  | | | |
| **20.** Is the household connected to: [a] a water distribution system [b]a water drainage system [c] an electricity system 0)no 1)yes |  | |  | |  |
| **21.** Where do you obtain the majority of your drinking water? 1)Purchase (containers) 2)Pipe inside the house 3)Pipe outside the house, but on the property 4)A communal pipe 5)River, spring 6)Well public or private 7)Water tanker 8)Rainwater 9)Other, what? | |  | | | |
| **22.** Where do you obtain the majority of the water for household use? 1)Pipe inside home 2)Pipe outside, but in yard 3)Pipe from public area (communal) 4)River, spring 5)Public or private, well 6)Tanker 7)Rainwater 8)Other, what? | |  | | | |
| **22b**. How far is the site where you collect water from the household? How long does it take to walk there? (meter/minute) | |  | |  | |
| **23.** Do you treat water before you drink it? If so, how? 1)No, none 2)Yes, boil. 3)Yes, filter. 4)Yes, chlorinate. 5)Yes, sodis 6)Other, what? | |  | | | |
| **24**. In the last month, has the household used firewood or sticks for cooking or other uses? 0)no 1)yes | |  | | | |
| **25**. Does the household collect firewood? 0)no 1)yes | |  | | | |
| **25b**. How far is the site where you collect firewood most frequently (from the household)? How long does it take to walk there? (meter/minute) | |  | |  | |
| **26.** In the last 5 years, the distance between the house and the site for firewood collection has? 1) Remained the same 2) Increased a Little 3) Decreased a Little 4) Increased a lot 5) Decreased a lot 6) Not sure | |  | | | |
| **27**. How did the household obtain firewood in the past month? 1) Purchased 2)Collected 3)Gifted/Given 6)Other, what? | |  | | | |

***II. EMPLOYMENT, ASSETS, AND INCOME***

| **1**. What is the principal occupation of men in this household? 1. Farmer 2. Rancher 3. Labourer 4. Independent 5. Carpenter 6. Marchant/sales 7. Mason 8. Teacher 9. Other? | | | |  | |
| --- | --- | --- | --- | --- | --- |
| **2**. What is the principal occupation of women in this family? 1. Housewife 2. Merchant 3. Farmer 4. Labourer 5. Teacher 6. Domestic worker 7. Other? | | | |  | |
| **3**. How many household members earn money? | | | |  | |
| **4**. In the last 12 months, how has the household obtained the majority of its money? | | | |  | |
| **4b.** Over the last 12 months, what has been the second most important source of money for the household? See Codes 1-19. | | | |  | |
| **5.**These sources of income are: 1. Stable throughout the year 2. Seasonal 3. Other, what? | | | |  |  |
| **6.** Has a member of the household received pay for work during the last 30 days? 0. No 1. Yes | | | |  | |
| **Name** | **# Days worked in the last month** | **Daily salary (quetzales)** | **Total (calculated)** | | |
|  |  |  |  |  | |
| **LOANS**  **7**a. Does the household currently have a debt to pay from a loan? 0. No 1. Yes | | | |  | |
| 7b. Who made the loan? | | | |  |  |
| 7c. What was the total amount of the loan? | | | |  | |
| 7d. How much does the household pay each month to pay off the loan? | | | |  | |

| **ASSETS**  **8.** Do you currently possess one of the following items in your household? **0. NO 1. YES** | | | | | | | |
| --- | --- | --- | --- | --- | --- | --- | --- |
| a. Refrigerator |  | c. Television |  | e. Radio |  | g. Cellphone |  |
| b. Bicycle |  | d. Motorcycle |  | f. Automobile |  | h. Fumigator sprayer |  |

| **OTHER SOURCES OF MONTHLY INCOME.** 9. How much did the family earn from these sources in the last month? | | | | | |  |
| --- | --- | --- | --- | --- | --- | --- |
| A. Family remittances |  | E. Sale of prepared food in an eatery |  | H. Sale of vegetables/crops from a family garden |  | |
| B. Artisanal sales |  | F. Mi Familia Progresa |  | I. Renting lands to other people |  | |
| C. Sale of herbs, wild forest products |  | G. Midwifery/ watching kids |  | J. Other (e.g., bonuses) |  | |
| D. Sale of hens and broilers |  |  |  |  |  | |

***III. FOOD SECURITY***

**AVAILABILITY**

| 1. In the last 12 months, has the households food production met household food needs? 1. Yes 2. No 3. Partially 4. Other, what? |  |
| --- | --- |
| 2. In the last 12 months, have you planted crops in a household garden? 0. No. 1. Yes. |  |
| 2b. In the last 12 months, what crops did you grow in the household garden? Use crop codes. |  |
| 3. In the last 12 months, the crops grown in the household garden have been for? 1. Household consumption 2. Local sale 3. International sale 4. Other, what? |  |

**CONSUMPTION**

| 4. In the last 12 months, has the household planted corn for your own consumption? 0. No 1. Yes |  |
| --- | --- |
| 5. Does the corn you harvest last for the whole year? 0. No 1. Yes |  |
| 6. In the last 7 days, how many pounds of corn have been cooked in the home? |  |
| 7. In a good season, how many pounds of corn are cooked in the home in one week? |  |
| 8. In a bad season, how many pounds of corn are cooked in the home in one week? |  |
| 9. Where does the corn that the household eats come from normally? 1. Own production and harvest 2. Purchased 3. Aid/gifted |  |
| 10. In the last 12 months, has the household planted bean for your own consumption? 0. No 1. Yes |  |
| 11. Does the bean you harvest last for the whole year? 0. No 1. Yes |  |
| 12. In the last 7 days, how many pounds of bean have been cooked in the home? |  |
| 13. In a good season, how many pounds of bean are cooked in the home in one week? |  |
| 14. In a bad season, how many pounds of bean are cooked in the home in one week? |  |
| 15. Where does the bean that the household eat come from normally? 1. Own production and harvest 2. Purchased 3. Aid/gifted. |  |

**ACCESS**

**The Household Food Insecurity Access Scale (HFIAS)**

|  | **Question** | **Option** | **Code** |
| --- | --- | --- | --- |
| **16.** | In the past four weeks, did you worry that your household would not have enough food? | 0 = No (skip); 1 = Yes |  |
| **16b.** | How often did this happen? |  |  |
| **17.** | In the past four weeks, were you or any household member not able to eat the kinds of foods you preferred because of a lack of resources? | 0 = No (skip); 1 = Yes |  |
| **17b.** | How often did this happen? |  |  |
| **18.** | In the past four weeks, did you or any household member have to eat a limited variety of foods due to a lack of resources? | 0 = No (skip); 1 = Yes |  |
| **18b.** | How often did this happen? |  |  |
| **19.** | In the past four weeks, did you or any household member have to eat some foods that you really did not want to eat because of a lack of resources to obtain other types of food? | 0 = No (skip); 1 = Yes |  |
| **19b.** | How often did this happen? |  |  |
| **20.** | In the past four weeks, did you or any household member have to eat a smaller meal than you felt you needed because there was not enough food? | 0 = No (skip)  1 = Yes |  |
| **20b.** | How often did this happen? |  |  |
| **21.** | In the past four weeks, did you or any other household member have to eat fewer meals in a day because there was not enough food? | 0 = No (skip)  1 = Yes |  |
| **21b.** | How often did this happen? |  |  |
| **22.** | In the past four weeks, was there ever no food to eat of any kind in your household because of a lack of resources to get food? | 0 = No (skip)  1 = Yes |  |
| **22b.** | How often did this happen? |  |  |
| **23.** | In the past four weeks, did you or any household member go to sleep at night hungry because there was not enough food? | 0 = No (skip)  1 = Yes |  |
| **23b.** | How often did this happen? |  |  |
| **24.** | In the past four weeks, did you or any household member go a whole day and night without eating anything because there was not enough food? | 0 = No (skip)  1 = Yes |  |
| **24b.** | How often did this happen? |  |  |

| **MONTHS OF ADEQUATE HOME FOOD PROVISIONING (MAHFP)** | | | | | | | | | |
| --- | --- | --- | --- | --- | --- | --- | --- | --- | --- |
| **25.** | In the past 12 months were there months in which you did not have enough food to meet your family’s needs? **0 = NO 1 = YES** | | | | | | **IF ANSWER IS NO, STOP HERE.** | | |
| **25b.** | If yes, which were the months (in the past 12 months) in which you did not have enough food to meet your family’s needs? 0. Enough 1. Not enough food | | | | | | | | |
| May | |  | February |  | November |  | | August |  |
| April | |  | January |  | October |  | | July |  |
| March | |  | December |  | September |  | | June |  |

**UTILIZATION AND HYGIENE**

| 26. Does the household use manure on its farm plots? 0. No 1. Yes |  |
| --- | --- |
| 27. Where do you normally store the manure prior to application? 1. In the agricultural parcel 2. Outside the home but on the immediate property 3. Inside the home 4. Other, what? |  |
| 28. Is there currently soap in the place where people wash their hands normally? 0. No 1. Yes |  |
| 29. In your household, how many flies are there currently? 1. Many 2. Some 3. Very few 4. None |  |
| 30. What do you do with household waste (usually)? 1. Throw it out 2. Burn it 3. Bury it 4. Sort and recycle it 5. Other, what? |  |

**STABILITY / VULNERABILITY**

| 31. In the last 12 months, have there been moments when the household has not had enough money to buy food or to cover other essentials? |  |
| --- | --- |
| 32. In the last 12 months, have you had to take one of the following actions to obtain food or satisfy other necessitities? |  |

| A. Looking for additional work, work longer hours |  | F. Reduce spending on fertilizers, pesticides, animal food |  | K. Eat less preferred food |  |
| --- | --- | --- | --- | --- | --- |
| B. Start a small business |  | G. Reduce health spending |  | L. Take children out of school so they can work. |  |
| C. Migrate elsewhere |  | H. Eat fewer times per day |  | M. Borrow food |  |
| D. Sell household possessions (e.g., television) |  | I. Reduce portion sizes for adults |  | N. Go entire days without eating |  |
| E. Sell animals more than usual (e.g., small animals) |  | J. Reduce portion sizes for children |  | O. Ask for aid from NGOs or other groups. Which groups? |  |

| 33. What have been the principal difficulties for the household in the last 12 months? For example, problems could relate to agriculture, prices, the envirionment, or jobs. **DO NOT LIST OPTIONS. SEE CODE LIST.** | **A** | **B** | **C** |
| --- | --- | --- | --- |
|  |  |  |  |
| 34. In the last 12 months, have you or members of your household received benefits such as (…)? **NO = 0 YES = 1** | | |  |
| 34. **(B)** Do you or members have your household presently receive (…)?  **NO = 0 YES = 1** | | |  |

| A. Microcredit loans |  | F. Money transfers for social assistance programs by NGOs or other groups |  | K. Free health services/medications |  |
| --- | --- | --- | --- | --- | --- |
| B. Free technical/agricultural extension services |  | G. School scholarships |  | L. Free hygiene supplies (e.g., soap) |  |
| C. Free seeds, fertilizers, or agricultural tools |  | H. Food for school programs (to consume at school or to take home) |  | M. Other assistance. Specify. |  |
| D. Metal lamina, wood, or other materials for home construction/repair |  | I. Free food ration for the home (e.g., for small children, lactating/pregnant mothers) |  |  |  |
| E. Mi Familia Progresa |  | J. Food for work |  | O |  |

| 35. Do you or a member of the household participate in a group or organization, for example: the church, a committee, communal agriculture groups, etc? **NO = 0 YES= 1** |  | |
| --- | --- | --- |
| 36. What groups do you participate in and/or are a member? |  | |
| 37. If suddenly you needed a small amount of money (for example, to pay forone weeks of spending), do ou believe that someone would help you to cover these costs? 1. *Definitely yes 2. Probably 3. Not sure 4. Probably not 5. Definitely not.* |  | |
| 38**.** If the household suffered an important economic loss, for example, a harvest loss, who do you believe would help you to fill/cover necessities? SEE CODE LIST. |  | |
| 39. In general, do you consider yourself happy? 1. Very happy 2. Partially happy 3. Neither happy nor unhappy 4. Somewhat unhappy 5. Not sure |  | |
| 40. Do you think you can change the future of your life? 1. Definitely yes 2. Probably 3. Not sure 4. Probably not 5. Definitely not 6. There are other people who have the power |  | |
| 41**.** Currently, what are the priority necessities to improve the well-being of your family? For example, priorities can be linked to: agriculture, health, prices, environment and/or work. SEE CODE LIST | **A** | **B** |
|  |  |  |

| **Product** | | **2.**  **COD.** | **COD.** | **QUET.** |  | **Product** | **2.**  **COD.** | **3.**  **COD.** | **4.**  **QUET.** |
| --- | --- | --- | --- | --- | --- | --- | --- | --- | --- |
| **A** | Bara de canastas |  |  |  | **B** | Hongos (ej. Orejo de pino, Oreja de gato, Oreja de burro, Silip) |  |  |  |
| **C** | Pamaque |  |  |  | **D** | Barretillo |  |  |  |
| **E** | Aciento |  |  |  | **F** | Mirto |  |  |  |
| **G** | Pino para canastas |  |  |  | **H** | Begonia silvestre |  |  |  |
| **I** | Musgo |  |  |  | **J** | Sangre de Cristo |  |  |  |
| **K** | Escasas orquídeas (parasitas) |  |  |  | **L** | Altamisa |  |  |  |
| **M** | Pacaya de ternera |  |  |  | **N** | Manzanillo |  |  |  |
| **O** | Macuy de montaña (hierba mora) |  |  |  | **P** | Apacin |  |  |  |
| **Q** | Mora silvestres |  |  |  | **R** | Arroyan |  |  |  |
| **S** | Pacaya disciplina |  |  |  | **T** | Ocote |  |  |  |
| **U** | Palmito de palma |  |  |  | **V** | Hierba de Danto |  |  |  |

**WILD PRODUCTS**

| 42. In the last 12 months, have you or a member of this household collected wild products from the forest, such as bara, aciento, blackberries, pacaya de ternera (or others) for any use (such as artisanry, eating, medicine)? **NO = 0 YES = 1** | **CODE** |
| --- | --- |
|  |  |

43. Specifically, in the last 12 months has someone collected (…) for the household? **NO = 0 YES = 1**

44. In the last 12 months, have some of the collected (…) been sold? **NO = 0 YES= 1**

45. In the last 12 months, what was the total amount of money received from selling (…)? **QUETZALES**

**UTILIZATION: HOUSEHOLD DIETARY DIVERSITY SCORE AND FOOD CONSUMPTION SCORE**

| Name of the mother | _____________ | Name, age, and sex of child | Name |  | Age |  | Sex |  |
| --- | --- | --- | --- | --- | --- | --- | --- | --- |

| **Household Dietary Diversity Score** | |  | **Food Consumption Score for children** For one randomly selected child, age 1-8 | |
| --- | --- | --- | --- | --- |
| 46. Was yesterday a special day where members of the household ate more or less than usual? **NO=0; YES = 1** | 47. Yesterday, did you or a member of your household eat (…) that was prepared in the home? NO =0; YES = 1  **EXCLUDE FOOD BOUGHT/PREPARED OUTSIDE THE HOME** |  | 48. In the last 7 days, how many days did this child eat (…)?  INCLUDE ALL SOURCES OF THE FOOD | 49. Where does the (…) this food for the child come from normally?  MARK THE MOST COMMON SOURCES. |
| **CODE** |  |  |  |  |
|  |  |  |  |  |

| **FOOD** | **47. COD** | **48. # OF DAYS** | **49. NORMAL SOURCE** |  | **47. COD** | **48. # OF DAYS** | **49. NORMAL SOURCE** | **Interviewer:** |
| --- | --- | --- | --- | --- | --- | --- | --- | --- |
| Corn tortillas, tamales, corn |  |  |  | Meat (chicken, beef, sausages, ham, etc) |  |  |  | Please use the following codes to register sources. If there is more than once source, list them from major to minor.   \| A – Own production / garden \| \| --- \| \| B – Bought in Chilascó \| \| C – Bought in Salamá \| \| D - Gifted/ \| \| E – Barter or trade \| \| F - Lent / debt \| \| G –Found in waste/ wild foods \| |
| Atoles de masa |  |  |  | Fish or seafood |  |  |  |  |
| Beans, peas, or peanuts |  |  |  | Acidic fruits (pineapple, lemon, orange) |  |  |  |  |
| Powdered milk, cheese, cream, or other dairy |  |  |  | Other fruits (papaya, mango, banana, melon, etc) |  |  |  |  |
| Oils, butter, margarine, avocado, |  |  |  | Vegetables (tomatoes, carrot, cabbage, squash, etc) |  |  |  |  |
| Eggs |  |  |  | Broccoli |  |  |  |  |
| Potatoes |  |  |  | Sodas |  |  |  |  |
| Plantains |  |  |  | Coffee, tea |  |  |  |  |
| Pacaya |  |  |  | Snacks (chips, etc.) |  |  |  |  |
| Bread |  |  |  | French fries, fried chicken |  |  |  |  |
| Rice |  |  |  | Sugar, candy, jams, honey, marshmallow |  |  |  |  |

***AGRICULTURE SURVEY***

***I. AGRICULTURAL PRODUCTION***

| 1. In the last month, have you planted corn for the household? **NO = 0 YES = 1** |  |
| --- | --- |
| 2. In the last 12 months, have you planted bean for the household? **NO = 0; YES= 1** |  |
| 3. In the last 12 months, have you planted broccoli for the household? **NO = 0; YES = 1** |  |
| 4. In what year did you start planting broccoli for the household? |  |
| 5. Have you planted broccoli for the household in the past? **NO = 0; YES = 1** |  |
| 6. When was the last year that you planted broccoli for the household? |  |
| 7. In the last 12 months, have you planted potato for the household? **NO = 0; YES = 1** |  |
| 8. In what year did you start planting potato for the household? |  |
| 9. Have you planted potato for the household in the past? **NO = 0; YES= 1** |  |
| 10 When was the last year that you planted potato for the |  |
| 11. How many years have you been a farmer? |  |
| 12. ASK IF APPLICABLE. 15 years ago, what crops did you plant on your land?  LIST THE FOUR MAJOR CROPS. MARK **00** IF DID NOT FARM. |  |
| 13. 10 years ago, what crops did you plant on your land?  LIST THE FOUR MAJOR CROPS. MARK **00** IF DID NOT FARM. |  |
| 14. 5 years ago, what crops did you plant on your land?  LIST THE FOUR MAJOR CROPS. MARK **00** IF DID NOT FARM. |  |
| 15. 2 years ago, what crops did you plant on your land?  LIST THE FOUR MAJOR CROPS. MARK **00** IF DID NOT FARM. |  |
| 16. What is the area of land that you currently own, rent, or rent out to others?  USE THE TENURE AND LOCATIONS CODES TO MARK TENURE TYPE AND LOCATION. |  |

|  | **AREA** | **TENURE** | **LOCATION** |  | **AREA** | **TENURE** |
| --- | --- | --- | --- | --- | --- | --- |
| A. Crops |  |  |  | D. Pasture/ livestocks |  |  |
| B. Natural forest |  |  |  | E. Fallow |  |  |
| C.Managed/artificial forest |  |  |  | F. Household land and patio |  |  |

| 17. Calculate the total land (in manzanas) |  |
| --- | --- |
| 18. Calculate the total land owned (in manzanas) |  |
| 19. Calculate the total rented land (in manzanas) |  |
| 20. Calculate the total land rented to others (in manzanas) |  |

**OWN (AGRICULTURAL PARCELS)**

| 21. In the last 12 months, have you worked in your own lands on behalf of the household? |  |
| --- | --- |
| 22. LIST THE PARCELS OR LOTS THAT YOU OWN INCLUDE PATIO LANDS (E.G. FAMILY GARDENS). |  |
| 23. What is the area of this parcel? |  |
| 24. What type of document credits ownership of this land to you? 1. Receipt 2. Deed 3. Deed recorded 4. Communal property tile 5. Do not have 6. Other, what? |  |
| 25. How long have you owned this parcel? |  |
| 26. How long does it take you to walk to this parcel from your home by foot? |  |
| 27. What do you consider is the quality of the soil on this land? 1. Good 2. Okay 3. Bad 4. Other |  |
| 28. How long has it been since you let this land lay fallow? |  |
| 29. What is the topography of the parcel? 1. Flat 2. Mostly flat 3. Ondulated 4. Steep 5. Very steep |  |
| 30. If you were to sell this parcel, how much could you receive for it? |  |

**RENTED (AGRICULTURAL PARCELS)**

| 31. In the last 12 months, have you worked in lands rented by the household? **NO = 0 YES = 1** |  |
| --- | --- |
| 32. **LIST THE PARCELS OR LOTS THAT YOU RENT FROM SOMEONE ELSE.** |  |
| 33. What is the area of this parcel? |  |
| 34. How long does it take you to walk to this parcel from your home by foot? |  |
| 35. What do you consider is the quality of the soil on this land?  1. Good 2. Okay 3. Bad 4. Other |  |
| 36. How long have you owned this parcel? |  |
| 37. What is the topography of the parcel? 1. Flat 2. Mostly flat 3. Ondulated 4. Steep 5. Very steep |  |
| 38. How do you pay to rent/work on this land? 1. Money 2. Harvest 3. Money and harvest 4. Work 5. Don’t pay 6. Other, specify. |  |
| 39. In the last 12 months, how much money have you paid in order to rent/use this land? |  |
| 40. Over the last 12 months, what quantity, if any, of the harvests did you have to give the property owner? |  |

**RENTED TO OTHERS (AGRICULTURAL PARCELS)**

| 41. In the last 12 months, have you rented land to others? **NO = 0 YES = 1** |  |
| --- | --- |
| 42. **LIST THE PARCELS OR LOTS THAT YOU RENT OUT TO SOMEONE ELSE.** |  |
| 43. What is the area of this parcel? |  |
| 44. In the last 12 months, how much money have you received as rent for this parcel? |  |
| 45. What crops the other farmer plant in the rented field in the last 12 months? |  |

**BOUGHT AND SOLD (AGRICULTURAL PARCELS) – LAST 12 MONTHS**

| 46. In the last 12 months, have you bought or sold land? NO = 0 YES = 1 |  |
| --- | --- |
| 47. LIST PARCELS BOUGHT OR SOLD IN THE LAST 12 MTHS |  |
| 48. (SOLD) What is the area of this parcel? |  |
| 49. (SOLD) How much money did you sell the land for |  |
| 50. (BOUGHT) What is the area of this parcel? |  |
| 51. (BOUGHT) How much money did you buy the land for? |  |

***II. AGRICULTURAL PRODUCTION***

| 1. **LIST THE NAMES OF ALL THE PARCELS.** |  |
| --- | --- |
| 2. In the last 12 months, what crops did you plant in this parcel? |  |
| 3. In the last 12 months, how many harvests of (…) did you have? |  |
| 4. In what months did you harvest (…)? |  |
| 5. In the first harvest from this parcel, how many quintales of (…) did you harvest? |  |
| 6. In the second harvest from this parcel, how many quintales of (…) did you harvest? |  |
| 7. In the third harvest from this parcel, how many quintales of (…) did you harvest? |  |
| 8. In the fourth harvest from this parcel, how many quintales of (…) did you harvest? |  |
| 9. What is the primary destination of production for this crop? 1. Household consumption 2 Local sale 3. Bring to Salama 4. Bring to Guatemala 5.Transformation 6. International export 7. Other |  |
| 10. Did this parcel contain broccoli, tomato, or potato in the last 12 months? **NO = 0 YES=1** **WHAT?** |  |
| 11. **LIST EVERY CROP FROM PREVIOUS.** How many quintales of (…) did you sell in the last 12 months? |  |
| 12. What was the total sale price of the (…)? |  |
| 13. How many quintales of (…) did you leave for household consumption? |  |
| 14. How many quintales of (…) wer lost or damaged before the harvest? |  |
| 15. How many quintales of (…) were left for animals? |  |
| 16. How many quin. of (…) did you leave for seed? |  |

***III. INPUTS AND EXPENDITURES***

**SEEDS**

| 1. Specify parcel number/name |  |
| --- | --- |
| 2. How much did you spend on seed or transplants for this parcel in the last 12 months? Specify by crop and price |  |
| 3. In the last 12 months, what types or varieties of corn did you plant in this parcel? 1. Yellow 2. White 3. Garden (Overo) 4. Mountain 5. Don’t farm corn |  |
| 4. In the last 12 months, what types of varieties of bean did you plant in this parcel? 1. Frijol del suelo 2. Frijol rallado 3. Frijol enredador 4. Piloy (negro) 5. Chui (amarillo) 6. Don’t farm bean 7. Other, specify. |  |
| 5. In the last 12 months, what broccoli companies did you work with in order to plant in this parcel? 1. MAYA-PAC/ Alcosa 2. Neo Alimentación 3. Legumex S.A. 4. Alimentos Sumar S.A. 5. Intermediary 6. Don’t farm broccoli 7. Other, specify |  |
| 6. In the last 12 months, what types or varieties of potato did you plant in this parcel? 1. Papa Toyoca 2. Papa Icta 3. Papa Loma 4. No potato 5. Other? |  |

**FERTILIZERS**

| 7. Specify parcel number/name |  |
| --- | --- |
| 8. In the last 12 months, have you applied chemical fertilizers to this parcel? |  |
| 9. How much triple quince (15-15-15) did you use in this parcel in the last 12 months (or per harvest)? |  |
| 10. How much urea (46-0-0) did you use in this parcel in the last 12 months (or per harvest)? |  |
| 11. How much veinte cero (20-20-0) did you use in this parcel in the last 12 months (or per harvest)? |  |
| 12. How much 15-0-25 did you use in this parcel in the last 12 months (or per harvest)? |  |
| 13. How much 18-8-12 did you use in this parcel in the last 12 months (or per harvest)? |  |
| 14. How much 27-0-12 did you use in this parcel in the last 12 months (or per harvest)? |  |
| 15. Specify parcel number/name |  |
| 16. How much (…) did you use in this parcel in the last 12 months (or per harvest)? A) Gallinaza cruda B) Ferti-organico C) Compost |  |
| 17. How much did you spend on (…) for this parcel in the last 12 months (or per harvest)? A) Gallinaza cruda B) Ferti-organico C) Compost |  |
| 18. In the last 12 months, what quantity of gallinaza from your own household did you use in this household? |  |
| 19. In the last 12 months, what quantity of compost from your own household did you use? |  |

**PESTICIDES**

| **20. PARCEL #** | 21.Please tell me if you used the following pesticide in the previous 12 months. If yes, what quantity did you use? | | | | | | | | | | | | |
| --- | --- | --- | --- | --- | --- | --- | --- | --- | --- | --- | --- | --- | --- |
|  | \| ***HERBICIDES***  1. GRAMOXONE*  2. GLIFOSATO*  3. RANGER*  ***FUNGICIDES***  4. AMBIL*  5. AMISTAR  6. ALTO*  7. CALDOBORDELÉS* \| 1. BRAVO 2. ROVRAL   10. METALAXYL  11. BELLIS  12. SILBACUR  **INSECTICIDES**  13. VOLATON  14. SEVIN  15. AVAUNT  16. RIENDA \| 17. TIODAN  18. ENDOSULFAN  19. TIODAN  20. MALATHION  21. LANNATE  22. GUSAFIN  23. ADMIRE, 24.CONFIDOR \| 25. SPINOACE, 26. SPINTOR  27. KRISOL  28. KARATE  29. ECOTECH  30. XENTARI  31. PERFEKTHION  32. DIBROM  33. DIBROXONE \| 34. PROCLAIM  35. MYCOTRAL  36. SERENADE  37. CLORPYRIFOS  38. TERBUFOS  39. AGROFOS  40. TERBUGRAN  41.CARBOFURAN  42. DIBROM \| 43.TAMARON  44.LORSBAN  45. AGROMIL  46.DIAZINON  47. BASUDIN  48. AMBUSH \| \| --- \| --- \| --- \| --- \| --- \| --- \| | | | | | | | | | | | | |
|  | **PRODUCT** | H1 | H2 | H3 | H4 | **TOTAL** | **PRODUCT** | H1 | H2 | H3 | H4 | | **TOTAL** |
|  |  |  |  |  |  |  |  |  |  |  |  |  | |

**EXPENDITURES**

| 22. In the last 12 months, how much did you spend in TOTAL on (…)? |  |
| --- | --- |

| ACTIVITY | TOTAL |  | TOTAL |  | TOTAL |
| --- | --- | --- | --- | --- | --- |
| A. Transport and freight payment |  | F. Production of agricultural sub-products |  | J. Fences and sheds |  |
| B. Product storage and drying |  | G. Gas and oils |  | K. Fees for veterinary services |  |
| C. Rental of agricultural machinery |  | H. Animal feed (e.g., corn, salt, concentrates, etc.) |  | L. Production of livestock by products |  |
| D. Maintenance and repair of machinery |  | I. Vaccines, remedies, or veterinary products |  | M. Agricultural labour – How many? What was the daily wage (GTQ/day)? |  |
| E. Rent working animals (for farm) |  |  |  |  |  |

**LIVESTOCK**

| 23. In the last 12 months, have you raised animals like chickens, ducks, goats, rabbits, pigs, cows, etc? | | | | | | |  | |
| --- | --- | --- | --- | --- | --- | --- | --- | --- |
| **NO** | **24. What animals**? | **NO = 0**  **YES = 1** | **25. How many (…) do you have currently?** | **26. How much could you sell 1 (…) for ?** | **27. In the last 12 months, how many (..) did you sell and at what price did you sell each one?** | | **28. In the last 12 months, how many of your (…) did the household consume?** | **29. How many (…) did you buy in the last 12 months?** |
| **1** | Bulls, cows or calves |  |  |  |  |  |  |  |
| **2** | Goats |  |  |  |  |  |  |  |
| **3** | Pigs |  |  |  |  |  |  |  |
| **4** | Rabbits |  |  |  |  |  |  |  |
| **5** | Fowl |  |  |  |  |  |  |  |
| **6** | Turkeys |  |  |  |  |  |  |  |
| **7** | Ducks |  |  |  |  |  |  |  |
| **8** | Horses or donkeys |  |  |  |  |  |  |  |
| **9** | Other, what? |  |  |  |  |  |  |  |

**ANIMAL SUB-PRODUCTS**

| 30. In the last 12 months, have you prepared a product from livestock? NO= 0; YES = 1  - Milk; Cheese; Egg; Honey; Leather; Butter; Wool; Sausage; Other, what? |  |
| --- | --- |
| 31. In the last 12 months, in total, how many (…) did you sell? |  |
| 32. For how much did you sell each unit? |  |

***IV. ENVIRONMENT***

| 1. Do you currently use any of the following practices on your agricultural land? **NO = 0 YES = 1** | | | | |  |
| --- | --- | --- | --- | --- | --- |
| Compost piles |  | Plant perpendicular to slope |  | Plant trees within the parcel |  |
| Bury organic matter to prepare the soil |  | Reforest |  | Plant fruit trees within the parcel |  |
| Plant vegetation along boundaries/fencelines |  | Allow land to rest fallow If so, how much time? ____________ |  | Use Gramaxone |  |
| Soil conservation using hedgerows |  | Use native seeds |  | Controlled burns prior to planting |  |
| Soil conservatoin using terraces |  | Use improved seeds |  | Management of natural regeneration |  |
|  |  | Harvest by the moon |  | Plant by the moon |  |

**PESTS**

| 2. . What crops are most affected by pests? 1. Corn 2. Bean 3. Broccoli 4. Tomato 5. Potato List in order of most to least. |  |
| --- | --- |
| 3. What pests/sicknesses most affect broccoli crops? 1. Gallina ciega 2. Plutell 3. Gallina ciega and Plutella .4. Others, what? |  |
| 4. Have you had problems with the fungus camotillo (clubroot) in your broccoli parcels? If so, in what parcel, since when, and why?  **NO = 0 YES=1** |  |
| **For he following questions, please answer if you are in agreement.** **NO = 0 YES= 1** |  |
| 5. Pest cause more damage to broccoli fields than to corn and bean fields |  |
| 6. There are more pests today than there were 5 years ago. |  |
| 7. There are insects that eat pests that eat the corn (who help control pests). |  |
| 8. There are insects that eat the pests of broccoli (who help control pests) |  |
| 9. Agricultural practices determine the levels of pests in fields. |  |

***V. INCOME AND WELL-BEING***

| 1. In the last 12 months, how have you obtained most of the money to support your family?  1b. What what the second most important source of money for your household? |  | | |  |
| --- | --- | --- | --- | --- |
| 2. In the last 12 months, how much money/income did your household have per month, on average? 1. Less than Q500 2. Q500 – Q1,000 3. Q1,000-Q2,000 4. Q2,000 – Q, 3000 5. More than Q3, 000 |  | | |  |
| 3. Compared with your household, other members of the community are in an economic position that is (…)?   1. Much better **2.** A little better 3. The same 4. A little worse 5. Much worse |  | | |  |
| 4. Before broccoli came to Chilascó, the economic situation in the community was: 1. Much better 2. A little better 3. Equal 4.A little worse 5. Much worse |  | | |  |
| 5. How do you see the change in Chilascó from farming broccoli? 1. Positive 2. Negative 3. The same |  | | |  |
| 6. Who benefits the most from the production of broccoli? 1. The household 2. The companies 3. The coyote 4. Everyone 5. Other, who? |  | | |  |
| Please state whether or not you agree with the following statements. **NO = 0 YES= 1** |  | | |  |
| 7. In Chilascó, there are big differences between the rich and the poor. |  | | |  |
| 8. The companies that export broccoli take advantage of the farmers. |  | | |  |
| 9. The companies that export broccoli never lose. |  | | |  |
| 10. The companies that export broccoli alway offer fair prices. |  | | |  |
| 11. A person who takes risks is better off economically. |  | | |  |
| 12. Does farming broccoli help you to feed your family? Why? 1. Yes 2. No 3. Yes and No 4. Other, what? |  | | |  |
| 13. In general do you consider yourself to be a happy person? How much? 1. Very happy 2. Partially happy 3. Neither happy nor unhappy 4. Somewhat unhappy 5. Very unhappy 6. Not sure |  | | |  |
| 14. Do you believe you have the power to change the future of your life? 1. Definitely yes **2.** Probably  **3.** Not sure **4.** Probably no **5.** Definitely no 6. Others have the power |  | | |  |
| 15. Presently, what are your priority needs in order to improve the wellbeing of your household? For example*,* priorities could relate to agriculture, health, prices,the environment, etc. SEE CODE LIST. |  |  |  | |

##

## HOUSEHOLD FOOD SECURITY SURVEY – SPANISH

| **FECHA (DD/MM/AA)** | ________/________/________ | **ENCUESTADOR** |  |
| --- | --- | --- | --- |

| **IDENTIFICACIÓN DEL HOGAR** | |
| --- | --- |
| 1. CAFESANO/PUESTO DE SALUD |  |
| 1. SECTOR No. |  |
| 1. HOGAR No. |  |
| 1. NÚMERO DE PERSONAS EN EL HOGAR |  |
| 1. NOMBRE DEL JEFE/A DEL HOGAR |  |
| 1. NOMBRE DE LA PARTICIPANTE |  |

Por favor, dos copias de la hoja de consentimiento informado tienen que ser firmadas por el participante antes de empezar la encuesta.

| **NÚMERO DE ENCUESTA**  **________/_________** | He completado el proceso de consentimiento informado con la participante y adjunto la carta firmada  **Sí No** |
| --- | --- |

**I. INFORMACIÓN GENERAL DEL HOGAR**

**A. MIEMBROS DEL HOGAR Y EDUCACIÓN**

|  | 1. ¿Cuál es el nombre del jefe o jefa del hogar que come y duerme habitualmente en este hogar? | 1. ¿Cuáles son los nombres, edad y sexo de todas las personas que comen y duermen normalmente en este hogar?   REGISTRE EL NOMBRE, EL SEXO, Y LA EDAD DE TODAS LAS PERSONAS QUE COMEN Y DUERMEN HABITUALMENTE EN ESTE HOGAR. INCLUYA A RECIEN NACIDOS Y ANCIANOS. | | | 1. ¿Qué nivel de estudios ha completado (...)? 2. Ninguno 3. Primaria (1 -3 grado) 4. Primaria (4- 6 grado) 5. Básico 6. Diversificado incompleto 7. Diversificado completo 8. Superior incompleto 9. Superior completo | 1. ¿(…) sabe leer y escribir? 2. Bien 3. Poco 4. No | 1. ¿Esta persona ha trabajado en tierras agrícolas por parte del hogar en los últimos 12 meses?   **0 =No**  **1 = Sí** |
| --- | --- | --- | --- | --- | --- | --- | --- |
| **NO** | **NOMBRE** | 1. **NOMBRE** | **(b) SEXO** | **(c) AÑOS** | **CÓDIGO** | **CÓDIGO** | **CÓDIGO** |
| **1** |  |  |  |  |  |  |  |
| **2** |  |  |  |  |  |  |  |
| **3** |  |  |  |  |  |  |  |
| **4** |  |  |  |  |  |  |  |
| **5** |  |  |  |  |  |  |  |
| **6** |  |  |  |  |  |  |  |
| **7** |  |  |  |  |  |  |  |
| **8** |  |  |  |  |  |  |  |
| **9** |  |  |  |  |  |  |  |
| **10** |  |  |  |  |  |  |  |
| **11** |  |  |  |  |  |  |  |
| **12** |  |  |  |  |  |  |  |

**B. TENENCIA DE LA VIVIENDA Y DE LA TIERRA**

| 1a. La vivienda que ocupa este hogar es (…):   1. Propia y totalmente pagada 2. Propia y pagándola a plazos 🡪 1B 3. Heredada o donada 4. Cedida o prestada 5. Alquilada 🡪 1B 6. Otra | 1b. ¿Cuánto paga mensualmente de alquiler? | 1. ¿Desde cuándo vive en la comunidad?   ANOTA EL AÑO EN QUE LLEGÓ. | 1. ¿Hay alguna persona en este hogar que se encuentre trabajando tierras agrícolas con producción propia?   **NO = 0 SÍ = 1** |
| --- | --- | --- | --- |
| **CÓDIGO** | **QUETZALES** | **A**Ñ**O DE LLEGADO** | **CÓDIGO** |
|  |  |  |  |

| 1. ¿Quién es la persona mejor informada sobre la actividad agrícola del hogar? | 1. ¿La entrevista se realiza con esta persona?   **NO = 0**  **SÍ = 1** | 1. ¿Cuál es el área de tierra agrícola que actualmente posee o ha arrendado?   1 mz = 6 cuerdas | 1. ¿El terreno donde cultiva es?   SI HAY MAS DE UN TIPO, FAVOR DE APUNTAR LOS TIPOS DE MAYOR A MENOR. | | 1. ¿Qué tipo de documento tiene que la acredita como propietaria de la tierra? | |
| --- | --- | --- | --- | --- | --- | --- |
|  |  |  | 1. Propio 2. Arrendado 3. Comunal 4. Privado | 1. Posesionario 2. Del Estado 3. Municipal 4. Asociación   /cooperativo | 1. Recibo 2. Escritura pública 3. Escritura registrada 4. Certificación municipal | 1. Título propiedad comunal 2. Otro, ¿cuál? 3. No tiene |
| **NOMBRE** | **CÓDIGO** | **MANZANAS** | **CÓDIGO** | | **CÓDIGO** | |
|  |  |  |  | |  | |

**C. ACCESO A VIVIENDA, AGUA Y ENERGIA**

|  | | | | **COD .** |
| --- | --- | --- | --- | --- |
| 1. ¿Cuál es el material predominante en las paredes exteriores de la casa? | 1. Block 2. Concreto 3. Madera (tabla) | | 1. Bajareque 2. Lamina metálica 3. Otro, ¿cuál? |  |
| 1. ¿De qué material es la mayor parte del techo? | 1. Lámina metálica 2. Tejas 3. Cemento | | 1. Paja, palma o similar 2. Otro, ¿cuál? |  |
| 1. ¿Cuál es el material predominante en el piso? | 1. Ladrillo de cemento o barro 2. Torta de cemento 3. Tierra, suelo | | 1. Piso cerámico 2. Madera 3. Otro, ¿cuál? ___________ |  |
| 1. ¿Cuál es el tipo de estufa? | 1. Estufa mejorada 2. Al suelo 3. Polleton | | 1. Estufa de gas propano 2. Estufa de gas keroseno 3. Otro, ¿cuál? ___________ |  |
| 1. ¿Qué tipo de servicio sanitario tiene este hogar? | 1. Inodoro conectado a red de drenajes 2. Letrina o pozo ciego | | 1. Excusado lavable 2. No tiene 3. Otro, ¿cuál? ___________ |  |
| 1. ¿Está conectada la vivienda a uno de los siguientes sistemas?   **0 = NO**  **1= SÍ** | A. | Una red de distribución de agua | |  |
|  | B. | Una red de drenajes | |  |
|  | C. | Una red de distribución de energía eléctrica | |  |

| 1. ¿De dónde obtienen **principalmente** el agua para beber? | | 1. ¿De dónde obtienen **principalmente** el agua para uso en el hogar (cocinar, lavar, etc)? | | 1. **(B)** ¿A qué distancia de su vivienda se encuentra el lugar de donde traen el agua y cuánto tiempo tarda? | | 1. ¿Le da tratamiento al agua antes de beber? | |
| --- | --- | --- | --- | --- | --- | --- | --- |
| 1. Comprada (garrafón) 2. Tubería, dentro de la vivienda 3. Tubería, fuera de la vivienda pero en el terreno 4. Tubería de un chorro comunal | 1. Río, manantial 2. Pozo público o privado 3. Camión cisterna 4. Agua de lluvia 5. Otro, ¿cuál? | 1. Tubería, dentro de la vivienda 2. Tubería, fuera de la vivienda pero en el terreno 3. Tubería de un chorro público (comunal) 🡪 **B** 4. Río, manantial 🡪 **B** | 1. Pozo público o privado 2. Camión cisterna 3. Agua de lluvia 4. Otro, ¿cuál? |  |  | 1. No, ninguno 2. Sí, la hierva 3. Sí, la filtra | 1. Sí, le pone cloro 2. Sí, sodis 3. Otro, ¿cuál? _____ |
| **CÓDIGO** | | **CÓDIGO** | | **(a) METR.** | **(b) MIN.** | **CÓDIGO** | |
|  | |  | |  |  |  | |

| 1. ¿Durante el mes pasado en este hogar, utilizaron leña o palos para cocinar o para otros usos?   **NO = 0 SÍ = 1** | 1. ¿Su hogar recolecta leña?   **NO = 0**  **SÍ = 1** | 1. **(B)** ¿A qué distancia de su casa queda el sitio de donde traen, o recogen o la leña con más frecuencia? ¿Cuánto tiempo tarda? | | 1. En los últimos 5 años, ¿la distancia entre su casa y el sitio de donde traen la leña: 2. Sigue igual 3. Ha aumentado un poco 4. Ha disminuido un poco 5. Ha aumentado mucho 6. Ha disminuido mucho 7. No estoy seguro/segura | 1. ¿Cómo obtuvo el hogar la leña que utilizó el mes pasado?   SI HAY MAS DE UNA FUENTE, FAVOR DE APUNTAR LAS FUENTES DE MAYOR A MENOR. | |
| --- | --- | --- | --- | --- | --- | --- |
|  |  |  |  |  | 1. Comprada 2. Recolectada 3. Regalada | 1. Otro, ¿cuál? |
| **CÓDIGO** | **CÓDIGO** | **METR.** | **MIN.** | **CÓDIGO** | **CÓDIGO** | |
|  |  |  |  |  |  | |

**II. TRABAJO Y ASPECTOS ECONÓMICOS**

| 1. ¿Cuál es la principal ocupación de los hombres de su familia? | | 1. Agricultor 2. Ganadero 3. Jornalero 4. Independiente 5. Carpintero | 1. Comerciante 2. Albañil 3. Maestro 4. Otro, ¿cuál? | | **COD.** |
| --- | --- | --- | --- | --- | --- |
|  |  |  |  |  |  |
| 1. ¿Cuál es la principal ocupación de las mujeres de su familia? | | 1. Ama de casa 2. Comerciante 3. Agricultora 4. Jornalera | 1. Maestra 2. Trabajadora doméstica 3. Otra, ¿cuál? | | **COD.** |
|  |  |  |  |  |  |
| 1. ¿Cuántos miembros del hogar ganan dinero? | **NÚMERO** | | | | |
|  |  | | | | |
| 1. ¿En los últimos 12 meses, cómo ha obtenido dinero principalmente dentro de su hogar?   4B. ¿Cuál ha sido la segunda fuente importante de dinero para su hogar?? | **(a) Fuente más importante** | | | **(b) Segunda fuente** | |
|  |  | | |  | |
| *NO MENCIONE LAS ALTERNATIVAS, PERMITA QUE LA PARTICIPANTE RESPONDA ESPONTANEAMENTE.*   1. Venta de la producción de granos básicos (maíz, frijol) 2. Venta de brócoli 3. Venta de papa 4. Venta de cultivos (en general) 5. Venta de animales/productos animales 6. Jornaleo en actividad agrícola **local** (empleado para labores agrícolas) 7. Jornaleo en actividad agrícola **migratorio** 8. Trabajo en la ‘Floresteria’ (producción de leatherleaf) 9. Trabajo no agrícola (guardián, trabajador doméstico, etc.) | 1. Trabajo independiente (chofer, carpintero, trabajador casual, etc.) 2. Empleado gubernamental (maestro, agente de salud, y administración) 3. Empleado de empresa privada u ONG 4. Venta de artesanías 5. Venta de productos silvestres (mora silvestre, etc.) 6. Pequeño comercio (pequeña escala) 7. Negocios (a mayor escala) 8. Subvenciones para el hogar, beneficios de desempleado 9. Remesas de emigrantes (dentro o fuera del país) 10. Otra (**especifique**) | | | | |

| 1. Esas fuentes de ingresos son: 2. Fijos durante todo el año 3. Estacional 4. Otro, ¿cuál? | **(a) Fuente más importante** | **(b) Segunda fuente** |
| --- | --- | --- |
|  |  |  |

| 1. ¿Algún miembro del hogar ha recibido pago por trabajo durante el último mes? **0=NO; 1=SI** | | | | | **CODIGO** |
| --- | --- | --- | --- | --- | --- |
|  |  |  |  |  |  |
| **Miembro del hogar** | | **Días trabajados en el último mes** | **Salario por día**  **(Quetzales)** | **Ingreso total por salarios (calculado)** | |
| **A.** |  |  |  |  | |
| **B.** |  |  |  |  | |
| **C.** |  |  |  |  | |
| **D.** |  |  |  |  | |
| **E.** |  |  |  |  | |

| **PRÉSTAMOS** | | | |  |
| --- | --- | --- | --- | --- |
| 1. **(a)** ¿ Tiene usted actualmente alguna deuda o préstamo que pagar?**0= No 1= Sí** | 1. **(b)** ¿Quién hizo el préstamo? | | 1. **(c)**¿Cuánto fue el monto del préstamo? | 1. **(d)** ¿Cuánto paga mensualmente por ese préstamo? |
|  | **A.** |  |  |  |
|  | **B.** |  |  |  |
|  | **C.** |  |  |  |

| **OTROS INGRESOS MENSUALES** | | | |
| --- | --- | --- | --- |
| 1. ¿Cuánto gano la familia por estas actividades en el último mes? | **QUETZALES** |  | **QUETZALES** |
| 1. Remesas familiares |  | 1. Mi Familia Progresa |  |
| 1. Venta de artesanías |  | 1. Atención de partos |  |
| 1. Venta de hierbas |  | 1. Venta de hortalizas cosechadas en un huerta familiar |  |
| 1. Venta chuntos, gallinas, pollos de engorde |  | 1. Arrendamiento de tierras |  |
| 1. Venta de alimentos en un comedor |  | 1. Otros Ingresos |  |
| **TOTAL** | | |  |

| **ACTIVOS** | | | |
| --- | --- | --- | --- |
| 1. ¿Tiene usted actualmente alguno de los siguientes productos en el hogar? **0 = NO ; 1 = SÍ** | | | |
| 1. Refrigerador |  | 1. Bicicleta |  |
| 1. Televisión |  | 1. Motocicleta |  |
| 1. Radio |  | 1. Automóvil, camión |  |
| 1. Teléfono celular |  | 1. Bomba de fumigar |  |

**III. SEGURIDAD ALIMENTARIA**

1. **DISPONIBILIDAD**

| 1. En los últimos 12 meses, ¿lo que cultivaron para la alimentación le alcanza? 2. Si 3. No 4. Parcialmente 5. Otro, ¿cuál? | 1. En los últimos 12 meses, ¿sembraron cultivos en un **huerto** **familiar**?   **NO = 0**  **SI = 1** | **2B.** En los últimos 12 meses, ¿qué cultivos cosecharon en este **huerto** familiar? | | | | | | | | 1. En los últimos 12 meses, lo que sembraron en el huerto familiar fue para: 2. Consumo familiar 3. Venta local 4. Venta internacional 5. Otra, ¿cuál?   SI HAY MAS DE UNA DESTINACIÓN, FAVOR DE APUNTARLAS DE MAYOR A MENOR. |
| --- | --- | --- | --- | --- | --- | --- | --- | --- | --- | --- |
|  |  | 1. Brócoli 2. Maíz 3. Frijol 4. Papa 5. Zanahoria 6. Tomate 7. Repollo 8. Rábano 9. Coliflor 10. Aguacate 11. Remolacha 12. Ejote | | | | 1. Güicoy 2. Güisquil 3. Lechuga 4. Apio 5. Duraznos 6. La hierba (macuy) 7. Cilantro 8. Chipilín 9. Acelga 10. Hierba buena 11. Medicinales 12. Otro, ¿cuál? | | | |  |
| **CÓDIGO** | **CÓDIGO** | **COD** | **COD** | **COD** | **COD** | | **COD** | **COD** | **COD** | **CÓDIGO** |
|  |  |  |  |  |  | |  |  |  |  |
|  |  |  |  |  |  | |  |  |  |  |

1. **CONSUMO**

| 1. En los últimos 12 meses, ¿han sembrado maíz por parte del hogar?   **NO = 0**  **SÍ = 1** | 1. ¿Lo que siembra de maíz le alcanza para todo el año?   **NO = 0**  **SÍ = 1** | 1. La semana pasada, ¿cuántas libras de maíz cocinó en el hogar? | 1. ¿En una buena época, cuántas libras de maíz cocina en una semana en el hogar? | 1. ¿En una mala época, cuántas libras de maíz cocina en una semana en el hogar? | 1. ¿De dónde viene el maíz que comen en su hogar normalmente? 2. Suyo/recogido 3. Comprado 4. Regalado   SI HAY MAS DE UNA FUENTE, FAVOR DE APUNTAR LAS FUENTES DE MAYOR A MENOR. | | |
| --- | --- | --- | --- | --- | --- | --- | --- |
| **COD.** | **COD.** | **LIBRAS** | **LIBRAS** | **LIBRAS** | **CÓDIGO.** | | |
|  |  |  |  |  |  |  |  |

| 1. En los últimos 12 meses, ¿han sembrado frijol por parte del hogar?   **NO = 0**  **SÍ = 1** | 1. ¿Lo que siembra de frijol le alcanza para todo el año?   **NO = 0**  **SÍ = 1** | 1. La semana pasada, ¿cuántas libras de frijoles cocinó en el hogar? | 1. ¿En una buena época, cuántas libras de frijoles cocina en una semana en el hogar? | 1. ¿En una mala época, cuántas libras de frijoles cocina en una semana en el hogar? | 1. ¿De dónde viene el frijol que comen en su hogar normalmente? 2. Suyo/recogido 3. Comprado 4. Regalado   SI HAY MAS DE UNA FUENTE, FAVOR DE APUNTAR LAS FUENTES DE MAYOR A MENOR. | | |
| --- | --- | --- | --- | --- | --- | --- | --- |
| **COD.** | **COD.** | **LIBRAS** | **LIBRAS** | **LIBRAS** | **CÓDIGO.** | | |
|  |  |  |  |  |  |  |  |

1. **ACESSO**

| **ESCALA DE ACCESO DE LA INSEGURIDAD ALIMENTARIA (HFIAS): 30 DIAS** | | | |
| --- | --- | --- | --- |
| **NO.** | **PREGUNTA** | **NO = 0 ; SÍ =1** | 1 = Pocas veces (una o dos veces)  2 = Algunas veces (entre tres y diez veces)  3 = Con frecuencia (más de diez veces) |
|  |  | **CÓDIGO** | **CÓDIGO DE FRECUENCIA** |
|  | En las últimas cuatro semanas, ¿le preocupó que en su hogar no hubiera suficientes alimentos? |  | - |
| 1B. | ¿Con qué frecuencia sucedió esto? |  |  |
|  | En las últimas cuatro semanas, ¿usted o algún miembro de la familia no pudo comer los tipos de alimentos preferidos debido a la falta de recursos? |  | - |
| 2B. | ¿Con qué frecuencia sucedió esto? | - |  |
|  | En las últimas cuatro semanas, ¿usted o algún miembro de la familia tuvo que comer una variedad limitada de alimentos debido a la falta de recursos? |  | - |
| 3B. | ¿Con qué frecuencia sucedió esto? | - |  |
|  | En las últimas cuatro semanas, ¿usted o algún miembro de la familia tuvo que comer alimentos que realmente no deseaba debido a la falta de recursos para obtener otros alimentos? |  | - |
| 4B. | ¿Con qué frecuencia sucedió esto? |  |  |
| 5. | En las últimas cuatro semanas, ¿usted o algún miembro de la familia tuvo que comer menos de lo que sentía que necesitaba porque no había suficientes alimentos? |  | - |
| 5B. | ¿Con qué frecuencia sucedió esto? |  |  |
| 6. | En las últimas cuatro semanas, ¿usted o algún miembro de la familia tuvo que comer menos veces por día porque no había suficientes alimentos? |  | - |
| 6B. | ¿Con qué frecuencia sucedió esto? | - |  |
| 7. | En las últimas cuatro semanas, ¿alguna vez no hubo absolutamente ningún alimento que comer en su hogar debido a la falta de recursos para adquirirlos? |  | - |
| 7B. | ¿Con qué frecuencia sucedió esto? | - |  |
| 8. | En las últimas cuatro semanas, ¿usted o algún miembro de la familia se fue a dormir por la noche con hambre porque no había suficientes alimentos? |  | - |
| 8B. | ¿Con qué frecuencia sucedió esto? | - |  |
|  | En las últimas cuatro semanas, ¿usted o algún miembro de la familia se pasó todo el día sin comer nada debido a que no había suficientes alimentos? |  |  |
| 9B. | ¿Con qué frecuencia sucedió esto? |  |  |

| **MESES DE APROVISIONAMIENTO ADECUADO DE ALIMENTOS (MAHFP)** | | **CÓDIGO** | | | |
| --- | --- | --- | --- | --- | --- |
|  | ¿En los últimos 12 meses, hubo meses en los que no tuvieron suficientes alimentos para satisfacer las necesidades de la familia? **0 = NO 1 = SÍ** | **SI RESPUESTA ES NO, PARA AQUÍ.** | | | |
| **10B.** | ¿Cuáles fueron los meses (en los últimos 12 meses) en los que no hubo suficientes alimentos para satisfacer las necesidades de la familia?  0 = HUBO SUFICIENTES ALIMENTOS  **1 = NO HUBO SUFICIENTES ALIMENTOS**  NO LEA LA LISTA DE MESES. REMONTÁNDOSE HACIA ATRÁS DESDE EL MES EN CURSO**, ESCRIBA UNO EN LA CASILLA SI LA PERSONA ENTREVISTADA IDENTIFICA DICHO MES**. | Mayo |  | Noviembre |  |
|  |  | Abril |  | Octubre |  |
|  |  | Marzo |  | Septiembre |  |
|  |  | Febrero |  | Agosto |  |
|  |  | Enero |  | Julio |  |
|  |  | Diciembre |  | Junio |  |

1. **UTILIZACIÓN**

| **HIGIENE** | | | |  |
| --- | --- | --- | --- | --- |
| 1. ¿Ustedes utilizan gallinaza en sus parcelas agrícolas?   **NO = 0 SÍ = 1** | 1. ¿Dónde guarda la gallinaza generalmente? 2. En la parcela agrícola 3. Fuera de la vivienda pero en el terreno 4. Dentro de la vivienda 5. Otro, ¿cuál? | 1. ¿Tiene jabón actualmente en el lugar donde lavan las manos normalmente?   **NO = 0 SÍ = 1** | 1. En su hogar, ¿cuántas moscas hay actualmente? 2. Muchas 3. Algunos 4. Muy pocos 5. Ninguno | 1. ¿Qué hacen ***principalmente*** con la basura del hogar? 2. La tiran 3. La queman 4. La entierran 5. La clasifican 6. Otro, ¿cuál? |
| **CÓDIGO** | **CÓDIGO** | **CÓDIGO** | **CÓDIGO** |  |
|  |  |  |  |  |

1. **ESTABILIDAD/VULNERABILIDAD**

| **ESTRATEGIAS DE SOBREVIVENCIA** | | | | | | |
| --- | --- | --- | --- | --- | --- | --- |
| 1. ¿Durante los últimos 12 meses, ha habido momentos cuando no ha tenido suficiente dinero para comprar alimentos o para cubrir otros gastos esenciales (salud, combustible para cocinar, escuela, etc.)?   **NO = 0 SÍ = 1** | PREGUNTA SI RESPONDIÓ **SÍ** A LA PREGUNTA ANTERIOR.     1. Durante los últimos 12 meses, ¿ha tenido que tomar alguna de las acciones siguientes para obtener alimentos y satisfacer otras necesidades?   **NO = 0 SÍ = 1** | | | | | |
| **CODIGO** |  | | **COD.** |  | | **COD.** |
|  | **A** | Buscar otros empleos, incrementar horas de trabajo |  | **I** | Disminuir la cantidad de porción de comidas para los adultos |  |
|  | **B** | Emprender pequeños negocios |  | **J** | Disminuir la cantidad de las porciones de comida de los niños(as) |  |
|  | **C** | Migrar a otros lugares |  | **K** | Comer alimentos menos preferidos |  |
|  | **D** | Vender posesiones del hogar (radio, muebles, televisión, etc.) |  | **L** | Retirar los niños(as) de las escuelas para trabajar |  |
|  | **E** | Vender animales más de lo acostumbrado (ej. menores) |  | **M** | Prestarse alimentos |  |
|  | **F** | Disminuir los gastos para fertilizantes pesticidas, alimentos para animales |  | **N** | Pasar días enteros sin comer |  |
|  | **G** | Disminuir los gastos en salud |  | **O** | Pedir ayuda a las instituciones   - ¿Cuáles? _____________________ |  |
|  | **H** | Comer un menor número de veces al día |  |  |  |  |

| 1. ¿Cuáles han sido sus principales dificultades o problemas en los últimos 12 meses? *Por ejemplo,* los problemas pueden ser relacionados a la agricultura, la salud, los gastos y precios, el medioambiente y/o fuentes de empleo.   *NO MENCIONE LAS ALTERNATIVAS, PERMITA QUE LA PARTICIPANTE RESPONDA ESPONTANEAMENTE Y LUEGO SOLICITE QUE PRIORICE LAS 3 MAS IMPORTANTES.* | **(a)** 1ra dificultad | **(b)** 2da dificultad | **(c)** 3ra dificultad |
| --- | --- | --- | --- |
|  | **CÓD.** | **CÓD.** | **CÓD.** |
| 1. Falta de empleo 2. Pérdida de empleo/salario reducido 3. Bajos precios de venta de brócoli 4. Bajos precios de venta de maíz/frijol 5. Pérdida de la cosecha de brócoli 6. Perdida de la cosecha de maíz/frijol 7. Altos precios de alimentos 8. Altos precios de combustible/transporte 9. Enfermedad/gastos de salud 10. Fallecimiento de miembros del hogar 11. Perdida de ayudas en dinero o especie 12. Reembolso de deuda 13. Agua para beber irregular/insegura 14. Problemas ambientales (contaminación, industrias) 15. Sequías, inundaciones, fuertes lluvias, deslizamientos de tierra 16. Disputa de tierras 17. Otro problema (especifique)   99- Si no menciona 2da o 3ra dificultad |  |  |  |

| **ASISTENCIA SOCIAL** | | 1. ¿Usted y los miembros del hogar recibieron en los últimos 12 meses beneficios tales como?   **NO = 0 SÍ = 1** | PREGUNTA SI RESPONDIÓ **SÍ** A LA PREGUNTA ANTERIOR.   1. **(B)** ¿Usted y los miembros del hogar reciben *actualmente* (…)?   **NO = 0 SÍ = 1** |
| --- | --- | --- | --- |
|  | Microcréditos |  |  |
|  | Asistencia técnica gratuita, servicios de extensión agrícola |  |  |
|  | Semillas, fertilizantes o herramientas agrícolas gratuitas (incluyendo programas de repago) |  |  |
|  | Laminas metálicas, madera, otro material para vivienda Mi Familia Progresa |  |  |
|  | Mi Familia Progresa |  |  |
|  | Transferencias de dinero de programas de asistencias social de ONGs, otras agencias |  |  |
|  | Becas escolares |  |  |
|  | Alimentación escolar (para consumir en la *escuela* o para llevar a casa) |  |  |
|  | Ración alimentaria gratuita para el *hogar* (ej. para niños pequeños/desnutridos o para mujeres lactantes/embarazadas) |  |  |
|  | Alimentos por *trabajo* |  |  |
|  | Atención de salud/medicamentos *gratuitos* |  |  |
|  | Artículos de higiene |  |  |
|  | Otro asistencia (especifique) |  |  |

| 1. ¿Usted o algún miembro del hogar es miembro o participa en algún grupo o organización, como por ejemplo: la iglesia, un comité, organizaciones comunales, organizaciones productivas o de cualquier otro tipo?   **NO = 0 SÍ = 1** | 6. ¿De qué grupo(s), organización(es) o asociación(es) es usted miembro o participante? | 7. Si repentinamente usted necesita una pequeña cantidad de dinero (por ejemplo, para pagar los gastos de una semana de su hogar), ¿cree usted que habría alguien que le ayudará para cubrir sus necesidades?   1. *Definitivamente sí* 2. *Probablemente* 3. *No está seguro* 4. *Probablemente no* 5. *Definitivamente no* | 8. Si su hogar sufriera una pérdida económica importante: por ejemplo: una pérdida de la cosecha, ¿quién cree usted que le ayudaría para cubrir sus necesidades? | |
| --- | --- | --- | --- | --- |
|  |  |  | REGISTRE LAS DOS PRIMERAS RESPUESTAS | |
|  |  |  | 1. *Nadie ayudaría* 2. *La familia* 3. *Los vecinos* 4. *Los amigos* 5. *Grupo religioso* 6. *Líder comunal* 7. *Líder empresarial / empresa* | 1. *Policía* 2. *El patrón/benefactor/ padrino* 3. *Un grupo u organización a la que pertenece* 4. *El gobierno* 5. *Una ONG* 6. *Otro, ¿cuál?* |
| **CÓDIGO** | **NOMBRE** | **CÓDIGO** | 1. **CÓDIGO** | 1. **CÓDIGO** |
|  |  |  |  |  |
|  |  |  |  |  |
|  |  |  |  |  |

| 1. ¿En general, se considera una persona feliz? | 1. ¿Cree usted que puede cambiar el futuro de su vida? | 1. Actualmente, ¿cuáles son sus necesidades prioritarias para mejorar el bienestar de su familia? *Por ejemplo,* las prioridades pueden ser relacionadas a la agricultura, la salud, los gastos y precios, el medioambiente y/o fuentes de empleo.   *REGISTRE LAS TRES PRIMERAS RESPUESTAS.* ***NO MENCIONE*** *LAS ALTERNATIVAS, PERMITA QUE LA PARTICIPANTE RESPONDA ESPONTANEAMENTE.* | | | |
| --- | --- | --- | --- | --- | --- |
| 1. Muy feliz 2. Feliz en parte 3. Ni feliz ni infeliz 4. Algo infeliz 5. Muy infeliz 6. No estoy seguro/a | 1. *Definitivamente sí* 2. *Probablemente* 3. *No está seguro* 4. *Probablemente no* 5. *Definitivamente no* 6. *Hay otras personas que tienen el poder* | 1. Alimentos para niños pequeños/desnutridos u otros grupos vulnerables 2. Alimentos para el hogar en general 3. Empleo 4. Transferencias de dinero 5. Crédito 6. Aumento de salario/pensión 7. Insumos agrícolas (semillas , fertilizantes, pesticidas) 8. Herramientas agrícolas | | 1. Forraje para animales 2. Riego 3. Agua para uso domestico 4. Servicios de salud (infraestructura, personal) 5. Carreteras 6. Escuela infraestructura/maestros 7. Vivienda/albergue (reparaciones, nuevo) 8. Combustible 9. Seguridad 10. Otra -especifique | |
|  |  |  |  |  |  |
| **CÓDIGO** | **CÓDIGO** | **(a) CÓDIGO** | **(b) CÓDIGO** | | **(c) CÓDIGO** |
|  |  |  |  | |  |

| **Producto** | | **2.**  **COD.** | **COD.** | **QUET.** |  | **Producto** | **2.**  **COD.** | **3.**  **COD.** | **4.**  **QUET.** |
| --- | --- | --- | --- | --- | --- | --- | --- | --- | --- |
| **A** | Bara de canastas |  |  |  | **B** | Hongos (ej. Orejo de pino, Oreja de gato, Oreja de burro, Silip) |  |  |  |
| **C** | Pamaque |  |  |  | **D** | Barretillo |  |  |  |
| **E** | Aciento |  |  |  | **F** | Mirto |  |  |  |
| **G** | Pino para canastas |  |  |  | **H** | Begonia silvestre |  |  |  |
| **I** | Musgo |  |  |  | **J** | Sangre de Cristo |  |  |  |
| **K** | Escasas orquídeas (parasitas) |  |  |  | **L** | Altamisa |  |  |  |
| **M** | Pacaya de ternera |  |  |  | **N** | Manzanillo |  |  |  |
| **O** | Macuy de montaña (hierba mora) |  |  |  | **P** | Apacin |  |  |  |
| **Q** | Mora silvestres |  |  |  | **R** | Arroyan |  |  |  |
| **S** | Pacaya disciplina |  |  |  | **T** | Ocote |  |  |  |
| **U** | Palmito de palma |  |  |  | **V** | Hierba de Danto |  |  |  |

1. **PRODUCTOS SILVESTRES**

| 1. En los últimos 12 meses, ¿recolectaron productos silvestres del bosque tales como bara, aciento, mora silvestres, pacaya de ternera (**u otros**) para hacer cualquier tipo de artesanías, para comer o para uso medicinal? **NO = 0 SÍ = 1** | **CODIGO** |
| --- | --- |
|  |  |

1. Específicamente, ¿en los últimos 12 meses han recogido (…) por parte del hogar? **NO = 0 SÍ = 1**
2. ¿En los últimos 12 meses, vendió parte del (…) recogido? **NO = 0 SÍ = 1**
3. ¿En los últimos 12 meses, cuál fue el monto total de la venta de (…)? **QUETZALES**

G. **UTILIZACION: Frecuencia de Consumo de Alimentos**

**
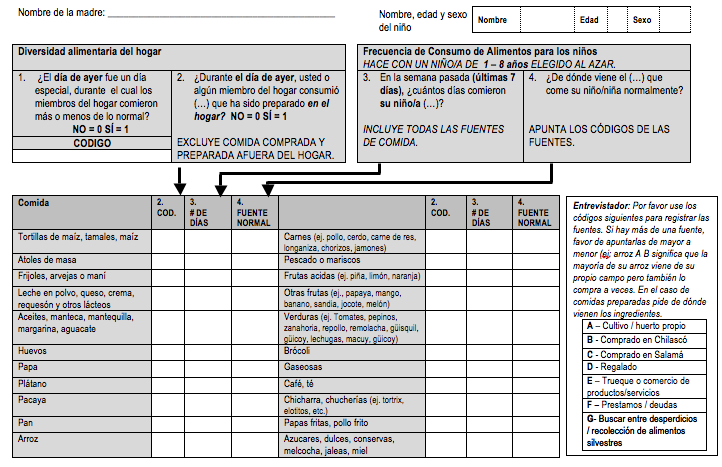
**

## AGRICULTURAL SURVEY – SPANISH

| **FECHA (DD/MM/AA)** | ________/________/________ | **ENCUESTADOR** |  |
| --- | --- | --- | --- |

| **IDENTIFICACIÓN DEL HOGAR** | |
| --- | --- |
| 1. CAFESANO/PUESTO DE SALUD |  |
| 1. SECTOR No. |  |
| 1. HOGAR No. |  |
| 1. NÚMERO DE PERSONAS EN EL HOGAR |  |
| 1. NOMBRE DEL JEFE/A DEL HOGAR |  |
| 1. NOMBRE DE LA PARTICIPANTE |  |

Por favor, dos copias de la hoja de consentimiento informado tienen que ser firmadas por el participante antes de empezar la encuesta.

| **NÚMERO DE ENCUESTA**  **________/_________** | He completado el proceso de consentimiento informado con la participante y adjunto la carta firmada  **Sí No** |
| --- | --- |

**SECCIÓN A. DATOS DE LA PRODUCCIÓN DE LA TIERRA**

| 1. ¿En los últimos 12 meses, ha sembrado maíz por parte del hogar?  **NO = 0**  **SÍ = 1** | 2. ¿En los últimos 12 meses, ha sembrado frijol por parte del hogar?  **NO = 0**  **SÍ = 1** | 3. ¿En los últimos 12 meses, ha sembrado brócoli por parte del hogar?  **NO = 0**  **SÍ = 1** | 4. ¿En qué año empezó a sembrar brócoli por parte del hogar? | PREGUNTA SI NO MENCIONÓ BROCOLI EN LA PREGUNTA ANTERIOR. | | 7. ¿En los últimos 12 meses, ha sembrado papa por parte del hogar?  **NO = 0**  **SÍ = 1** | 8. ¿En qué año empezó a sembrar papa por parte del hogar? | PREGUNTA SI NO MENCIONÓ PAPA EN LA PREGUNTA ANTERIOR | | |
| --- | --- | --- | --- | --- | --- | --- | --- | --- | --- | --- |
|  |  |  |  | 5. ¿Ha sembrado brócoli por parte del hogar en el pasado?  **NO = 0**  **SÍ = 1** | 6. ¿Cuál fue el último año que sembró brócoli? |  |  | 9. ¿Ha sembrado papa por parte del hogar en el pasado?  **NO = 0**  **SÍ = 1** | 10. ¿Cuál fue el último año que sembró papa? | |
| **CODIGO** | **CODIGO** | **CODIGO** | **AÑO** | **CODIGO** | **AÑO** | **CODIGO** | **AÑO** | **CODIGO** | | **AÑO** |
|  |  |  |  |  |  |  |  |  | |  |

| 11. ¿Hace cuántos años usted ha sido agricultor? | 12. PREGUNTA SI APLICA.  Hace 15 años, ¿qué cultivos sembraba en su tierra?  PRIORICE LOS 4 CULTIVOS DOMINANTES.  *ANOTA 00, SI NO SEMBRÓ.* | | | | | 13. Hace 10 años, ¿qué cultivos sembraba en su tierra?  PRIORICE LOS 4 CULTIVOS DOMINANTES.  *ANOTA 00, SI NO SEMBRÓ.* | | | | | | 14. Hace 5 años, ¿qué cultivos sembraba en su tierra?  PRIORICE LAS 4 CULTIVOS DOMINANTES.  *ANOTA 00, SI NO SEMBRÓ.* | | | | | 15, Hace 2 años, ¿qué cultivos sembraba en su tierra?  PRIORICE LAS 4 CULTIVOS DOMINANTES.  *ANOTA 00, SI NO SEMBRÓ,* | | | | |  | | CULTIVOS   1. Brócoli 2. Maíz 3. Frijol 4. Papa 5. Arveja 6. Zanahoria 7. Tomate 8. Repollo 9. Rábano 10. Coliflor 11. Aguacate 12. Remolacha |
| --- | --- | --- | --- | --- | --- | --- | --- | --- | --- | --- | --- | --- | --- | --- | --- | --- | --- | --- | --- | --- | --- | --- | --- | --- |
| **ANOS** | **CULTIVO** | | | | | **CULTIVO** | | | | | | **CULTIVO** | | | | | **CULTIVO** | | | | |  | | 13. Ejote |
|  |  |  | |  |  |  | |  |  | |  |  |  | |  |  |  |  | |  |  |  | | 99. Otro, ¿cuál? |
| 16. ¿Cuál es el área de terreno que actualmente tiene, ha arrendado, o dado en arrendamiento? | | | | | | | | | | | | | | | | | | | | | | | | |
| \| UTILIZA LOS CÓDIGOS DE TENENCIA SIGUIENTES:   1. Propio 2. Arrendado 3. Dada en arrendamiento 4. Otro, especifique \|  \| UTILIZA LOS CÓDIGOS DE UBICACIÓN SIGUIENTES:   1. Finca Chilascó 2. Finca San Antonio 3. Otro, especifique \| \| --- \| --- \| --- \| | | | | | | | | | | | | | | | | | | | | | | | | |
|  | | | **TAMAÑO** | | | | **TENENCIA** | | | **UBICACIÓN** | | | |  | | | | | **TAMAÑO** | | | | **TENENCIA** | |
| A. Cultivos | | |  | | | |  | | |  | | | | D. Potreros / Dedicada a la ganadería | | | | |  | | | |  | |
|  |  |  |  | | | |  | | |  | | | |  |  |  |  |  |  |  |  |  |  |  |
|  |  |  |  | | | |  | | |  | | | |  |  |  |  |  |  |  |  |  |  |  |
|  |  |  |  | | | |  | | |  | | | |  |  |  |  |  |  |  |  |  |  |  |
|  |  |  |  | | | |  | | |  | | | |  |  |  |  |  |  |  |  |  |  |  |
| B. Bosque natural | | |  | | | |  | | |  | | | | E. Tierra ociosa / cansada por cultivos | | | | |  | | | |  | |
| C. Bosque artificial/manejado | | |  | | | |  | | |  | | | | F. Tierra para la vivienda | | | | |  | | | |  | |

| **CONVERSION DE UNIDADES** | | |  | **17. TOTAL DE TIERRA** | **18. TOTAL TIERRA PROPIO** | **19. TOTAL TIERRA ARRENDADO** | **20. TOTAL TIERRA DADA EN ARRENDAMIENTO** |
| --- | --- | --- | --- | --- | --- | --- | --- |
|  |  |  |  | **MANZ** | **MANZ** | **MANZ** | **MANZ** |
| 1 manzana | =  = | 6 de a veinte (6 tareas de 20 brazadas)  0.7 hectáreas (7,000 m^2^) |  |  |  |  |  |
| 1 cuerda | = | 1 tarea (~ 1 de a veinte) |  |  |  |  |  |
| SI DIGA TAREA, PREGUNTA CUÁNTOS DE A VEINTES. | | |  |  |  |  |  |

| **TERRENOS O PARCELAS PROPIAS** | | | | | | | | | | |
| --- | --- | --- | --- | --- | --- | --- | --- | --- | --- | --- |
| 1. ¿En los últimos 12 meses, ustedes trabajaron en terrenos **propios** por parte del hogar? | | | | SÍ -🡪 ¿En cuántas fincas? ______ NO🡪 PASA A PROXIMA SECCION | | | | | | |
| 2. REGISTRE LOS TERRENOS, PARCELAS O LOTES DONDE TIENEN LAS TIERRAS PROPIAS. **INCLUYA A TIERRAS DEL PATIO EN LAS QUE TIENEN SIEMBRAS (ej. HUERTOS FAMILIARES)** | | 3. ¿Cuánto mide esta parcela? | 4. ¿Qué tipo de documento tiene que la acredita como propietaria de la tierra? | | 5. ¿Hace cuántos años que usted tiene esta terreno o parcela? | 6. ¿Cuánto tiempo necesita para llegar a este parcela a pie? | 7. ¿Cómo considera la fertilidad de los suelos de esta parcela?   1. Buena 2. Regular 3. Mala 4. Otro | 8.¿Hace cuánto tiempo que no ha sembrado este terreno? | 9. ¿Cómo es el terreno de esta parcela?   1. Plano 2. Semiplano 3. Ondulado 4. Quebrado 5. Muy quebrado | 10. Si fuera a vender esta parcela, ¿en cuánto la puede vender? |
|  |  |  | 1. Recibo 2. Escritura pública 3. Escritura registrada 4. Título propiedad comunal 5. No tiene 6. Otro, ¿cuál? | |  |  |  |  |  |  |
| **#** | **NOMBRE** | **MANZ.** | **CÓDIGO** | | **AÑOS** | **MIN.** | **CODIGO** | **MESES** | **CODIGO** | **QUET.** |
| **1** |  |  |  | |  |  |  |  |  |  |
| **2** |  |  |  | |  |  |  |  |  |  |
| **3** |  |  |  | |  |  |  |  |  |  |
| **4** |  |  |  | |  |  |  |  |  |  |
| **5** |  |  |  | |  |  |  |  |  |  |
| **6** |  |  |  | |  |  |  |  |  |  |

| **FINCAS O PARCELAS ARRENDADAS** | | | | | | | | | | |
| --- | --- | --- | --- | --- | --- | --- | --- | --- | --- | --- |
| 11. ¿En los últimos 12 meses, ustedes trabajaron en tierras arrendadas por parte del hogar? **NO = 0 SÍ = 1** | | | | | | | | | **CÓDIGO** | |
|  |  |  |  |  |  |  |  |  |  | |
| **12. REGISTRE LOS NOMBRES DE LAS FINCAS CON LAS TIERRAS EN ARRIENDO.** | | 13. ¿Cuánto mide esta parcela? | 14. ¿Cuánto tiempo necesita para llegar a esta parcela a pie? | 15. ¿Cómo considera la fertilidad de los suelos de esta parcela?   1. Buena 2. Regular 3. Mala 4. Otro | 16. ¿Hace cuántos años que usted tiene esta finca o parcela? | 17. ¿El terreno de esta parcela es?   1. Plano 2. Semiplano 3. Ondulado 4. Quebrado 5. Muy quebrado | 18. ¿Por trabajar en este parcela, usted paga en?   1. Dinero 2. Cosecha 3. Cosecha y dinero 4. Trabajo 5. No paga 6. Otro, ¿cuál? | 19. ¿Cuánto dinero ha pagado por el uso de esta parcela en los últimos 12 meses? | 20. ¿Qué cantidad de la cosecha de esta parcela ha tenido que dar al propietario por el uso de la tierra en los ultimos 12 meses? | |
| **#** | **NOMBRE** | **MANZ.** | **MIN.** | **CODIGO** | **AÑOS** | **CÓDIGO** | **CÓDIGO** | **QUET.** | **COD de CULT** | **QQ** |
| **1** |  |  |  |  |  |  |  |  |  |  |
| **2** |  |  |  |  |  |  |  |  |  |  |
| **3** |  |  |  |  |  |  |  |  |  |  |
| **4** |  |  |  |  |  |  |  |  |  |  |
| **5** |  |  |  |  |  |  |  |  |  |  |

| **TERRENOS DADAS EN ARRENDAMIENTO** | | | | | | | | |
| --- | --- | --- | --- | --- | --- | --- | --- | --- |
| 21. En los últimos 12 meses, ¿ustedes han dado tierra en arrendamiento?  **NO = 0 SÍ = 1** | | | | | | | | **CÓDIGO** |
|  |  |  |  |  |  |  |  |  |
|  | 22. REGISTRE EN CADA FILA EL NOMBRE DE LOS TERRENOS DADAS EN ARRENDAMIENTO. | 23. ¿Cuánto mide el terreno que ha dado en arrendamiento? | 24. ¿Cuánto dinero ha recibido usted para el uso de este terreno en los últimos 12 meses? | | | | | 25. ¿Qué cultivos estuvieron sembrados por parte del otro agricultor en este terreno? |
|  |  |  | Cos1 | Cos2 | Cos3 | Cos4 | **TOTAL** |  |
| # | NOMBRE | MANZANAS | QUETZALES | | | | | **CULTIVO** |
|  |  |  |  |  |  |  |  |  |
|  |  |  |  |  |  |  |  |  |
|  |  |  |  |  |  |  |  |  |
|  |  |  |  |  |  |  |  |  |

| **TERRENOS O PARCELAS VENDIDAS O COMPRADAS EN LOS ÚLTIMOS 12 MESES** | | | | | |
| --- | --- | --- | --- | --- | --- |
| 26. En los últimos 12 meses, ¿vendió o compró algún terreno o parcela? **NO = 0 SÍ = 1** | | | | **CÓDIGO** | |
|  |  |  |  |  | |
|  | 27. REGISTRE EN CADA FILA EL NOMBRE DE LOS TERRENOS VENDIDOS O COMPRADOS POR EL HOGAR. | **VENDIDAS** | | **COMPRADAS** | |
|  |  | 28. ¿Cuánto mide el terreno que vendió? | 29. ¿En cuánto dinero la vendieron? | 30. ¿Cuánto mide el terreno que compró? | 31. ¿En cuánto dinero la compraron? |
| # | NOMBRE | MANZANAS | QUETZALES | MANZANAS | QUETZALES |
| **1** |  |  |  |  |  |
| **2** |  |  |  |  |  |
| **3** |  |  |  |  |  |
| **4** |  |  |  |  |  |
| **5** |  |  |  |  |  |

**SECCIÓN B. PRODUCCIÓN AGRÍCOLA**

| **#** | **1. ESCRIBA LOS NOMBRES DE TODAS LAS PARCELAS DE LAS ÚLTIMAS SECCIONES.** | 2. ¿Qué cultivos ha cultivado en esta parcela en los últimos 12 meses? | 3. ¿Cuántas cosechas de (…) sacó en los últimos 12 meses de esta parcela? | 4. ¿En qué meses sacó las cosechas de esta parcela? | | | | 5. ¿Cuántas quintales de (…) sacó en la primera cosecha de esta parcela? | 6. ¿Cuántas quintales de (…) sacó en la segunda cosecha de esta parcela? | 7. ¿Cuántas quintales de (…) sacó en la tercera cosecha de esta parcela? | 8. ¿Cuántas quintales de (…) sacó en la cuarta cosecha de esta parcela? | 9. ¿Cuál es el destino principal de la producción de este cultivo?   1. Consumo en el hogar 2. Vender a mercado local 3. Lo lleva a Salamá 4. Lo lleva a Guatemala 5. Transformación en producto 6. Exportación internacional 7. Otro, ¿cuál? | 10. ¿Esta parcela ha tenido brócoli, papa o tomate en los últimos 12 meses?    **NO = 0 SÍ=1**  **ESPECIFIQUE** |
| --- | --- | --- | --- | --- | --- | --- | --- | --- | --- | --- | --- | --- | --- |
|  |  | **CULTIVO** | **NÚMERO** | **MESES** | | | | **QUINTALES** | **QUINTALES** | **QUINT** | **QUINT** | **CÓDIGO** | **CÓDIGO** |
|  |  |  |  |  |  |  |  |  |  |  |  |  |  |
|  |  |  |  |  |  |  |  |  |  |  |  |  |  |
|  |  |  |  |  |  |  |  |  |  |  |  |  |  |
|  |  |  |  |  |  |  |  |  |  |  |  |  |  |
|  |  |  |  |  |  |  |  |  |  |  |  |  |  |
|  |  |  |  |  |  |  |  |  |  |  |  |  |  |
|  |  |  |  |  |  |  |  |  |  |  |  |  |  |
|  |  |  |  |  |  |  |  |  |  |  |  |  |  |
|  |  |  |  |  |  |  |  |  |  |  |  |  |  |
|  |  |  |  |  |  |  |  |  |  |  |  |  |  |
|  |  |  |  |  |  |  |  |  |  |  |  |  |  |
|  |  |  |  |  |  |  |  |  |  |  |  |  |  |

| **ESCRIBA CADA CULTIVO DE LA SECCION ANTERIOR. .** | | 11. ¿Cuántos quintales de (…) vendió durante los últimos 12 meses? | | | | 12. ¿Cuál fue el monto total de la venta de (…)? | | | | 13. ¿Cuántos quintales de (…) se dejó para el consumo de la casa? | | | | 14. ¿Cuántos quintales de (…) se perdió o se malogró antes de la cosecha? | | | | 15. ¿Cuántos quintales de (…) se dejó para el alimento o forraje de los animales? | | 16. ¿Cuántos quintales de (…) se dejó para semilla? |
| --- | --- | --- | --- | --- | --- | --- | --- | --- | --- | --- | --- | --- | --- | --- | --- | --- | --- | --- | --- | --- |
|  |  | **QUINTALES** | | | | **QUETZALES** | | | | **QUINTALES** | | | | **QUINTALES** | | | | **QUIN** | | **QUIN** |
| **#** | **CUL.** | **Cos1** | **Cos2** | **Cos3** | **TOT** | **Cos1** | **Cos2** | **Cos3** | **TOT** | **Cos1** | **Cos2** | **Cos3** | **TOT** | **Cos1** | **Cos2** | **Cos3** | **TOT** | **TOT** | **TOTAL** | |
|  |  |  |  |  |  |  |  |  |  |  |  |  |  |  |  |  |  |  |  | |
|  |  |  |  |  |  |  |  |  |  |  |  |  |  |  |  |  |  |  |  | |

**SECCIÓN C. INSUMOS**

| **SEMILLAS** | | | | | | | | | | | |
| --- | --- | --- | --- | --- | --- | --- | --- | --- | --- | --- | --- |
| **#** | **1. NOMBRE DE LA PARCELA** | 2. ¿Cuánto gasto en semillas y/o pilones para esta parcela en los últimos 12 meses (o por cosecha)? | | | | | | 3. En los últimos 12 meses, ¿qué tipos o variedades de semillas de maíz ha utilizado en esta parcela?   1. Amarilla 2. Blanca 3. Negra 4. De huerta (Overo) 5. De montaña 6. No sembró maíz   ANOTA CADA TIPO | 4. En los últimos 12 meses, ¿qué tipos o variedades de semillas de frijol ha utilizado en esta parcela?   1. Frijol del suelo 2. Frijol rallado 3. Frijol enredador 4. Piloy (negro) 5. Chui (amarillo) 6. No sembró frijol 7. Otro, ¿cuál? | 5. En los últimos 12 meses, ¿con que empresas de brócoli ha trabajado este terreno?   1. MAYA-PAC/ Alcosa 2. Neo Alimentación 3. Legumex S.A. 4. Alimentos Sumar S.A. 5. Con un coyote / intermediario 6. No sembró brócoli 7. Otra, ¿cuál?   ANOTA CADA EMPRESA | 6. En los últimos 12 meses, ¿qué tipos o variedades de semilla de papa ha utilizado?   1. Papa Toyoca 2. Papa Icta 3. Papa Loma 4. No sembró papa 5. Otro, ¿cuál? |
|  |  | **CULT** | Cos1 | Cos2 | Cos3 | Cos4 | **TOT** |  |  |  |  |
|  |  | **QUETZALES** | | | | | | **CODIGO** | **CODIGO** | **CÓDIGO** | **CODIGO** |
|  |  |  |  |  |  |  |  |  |  |  |  |
|  |  |  |  |  |  |  |  |  |  |  |  |
|  |  |  |  |  |  |  |  |  |  |  |  |
|  |  |  |  |  |  |  |  |  |  |  |  |
|  |  |  |  |  |  |  |  |  |  |  |  |
|  |  |  |  |  |  |  |  |  |  |  |  |

| **FERTILIZANTES** | | | | | | | | | | | | | | | | | | | | |
| --- | --- | --- | --- | --- | --- | --- | --- | --- | --- | --- | --- | --- | --- | --- | --- | --- | --- | --- | --- | --- |
| **#** | **7. NOMBREDE LA PARCELA** | 8. En los últimos 12 meses, ¿utilizó fertilizantes químicos en esta parcela? | ¿Cuánto (…) utilizó en esta parcela en los últimos 12 meses (o por cosecha)?  ANOTA 00, SI NO UTILIZÓ  ESPECIFIQUE LOS UNIDADES. | | | | | | | | | | | | | | | | | |
|  |  |  | **9.** | | | | | **10.** | | | | | **11.** | | | | | **12.** | **13.** | **14.** |
|  |  |  | **Triple quince (15-15-15)** | | | | | **Urea**  **(46-0-0)** | | | | | **Veinte cero (20-20-0)** | | | | | **15-0-25** | **18-8-12** | **27-0-12** |
|  |  | **CÓDIGO** | **Cos1** | **Cos2** | **Cos3** | **Cos4** | **TOTAL** | **Cos1** | **Cos2** | **Cos3** | **Cos4** | **TOTAL** | **Cos1** | **Cos2** | **Cos3** | **Cos4** | **TOTAL** | **TOTAL** | **TOTAL** | **TOTAL** |
|  |  |  |  |  |  |  |  |  |  |  |  |  |  |  |  |  |  |  |  |  |
|  |  |  |  |  |  |  |  |  |  |  |  |  |  |  |  |  |  |  |  |  |
|  |  |  |  |  |  |  |  |  |  |  |  |  |  |  |  |  |  |  |  |  |

| **#** | **15. NOMBR DE LA PARCELA** | 16. ¿Cuánto (…) utilizó en esta parcela en los últimos 12 meses (o por cosecha)?  17. ¿Cuánto gastó en (…) para esta parcela en los últimos 12 meses (o por cosecha)?  ANOTA 00, SI NO UTILIZÓ  ESPECIFIQUE LOS UNIDADES | | | | | | | | | | | | | 18. En los últimos 12 meses, ¿qué cantidad de gallinaza de su propio hogar utilizó en esta parcela? | 19. En los últimos 12 meses, ¿qué cantidad de compost de su propio hogar utilizó en esta parcela? |
| --- | --- | --- | --- | --- | --- | --- | --- | --- | --- | --- | --- | --- | --- | --- | --- | --- |
|  |  |  | **a) Gallinaza cruda** | | | | **b) Ferti- orgánico** | | | | **c) Compost** | | | |  |  |
|  |  |  | **Cos1** | **Cos2** | **Cos3** | **TOTAL** | **Cos1** | **Cos2** | **Cos3** | **TOTAL** | **Cos1** | **Cos2** | **Cos3** | **TOT** | **CANTIDAD** | **CANTIDAD** |
|  |  | **A. CANT** |  |  |  |  |  |  |  |  |  |  |  |  |  |  |
|  |  | **B. QUET** |  |  |  |  |  |  |  |  |  |  |  |  |  |  |

| **PLAGUICIDAS** | | | | | | | |  | | | | | | |
| --- | --- | --- | --- | --- | --- | --- | --- | --- | --- | --- | --- | --- | --- | --- |
| **#** | **20. NOMBRE DE LA PARCELA** | 21. Por favor, dígame la cantidad de cada tipo de plaguicidas que utilizó en esta parcela en los últimos 12 meses. | | | | | |  | | | | | | |
|  |  | \| ***POR FAVOR, UTILIZA LOS CÓDIGOS SIGUIENTES.*** \| \| \| \| \| \| \| --- \| --- \| --- \| --- \| --- \| --- \| \| ***HERBICIDAS***  1. GRAMOXONE*  2. GLIFOSATO*  3. RANGER*  ***FUNGICIDAS***  4. AMBIL*  5. AMISTAR  6. ALTO*  7. CALDO-BORDELÉS* \| 1. BRAVO 2. ROVRAL   10. METALAXYL  11. BELLIS  12. SILBACUR  **INSECTICIDAS**  13. VOLATON  14. SEVIN  15. AVAUNT  16. RIENDA \| 17. TIODAN  18. ENDOSULFAN  19. TIODAN  20. MALATHION  21. LANNATE  22. GUSAFIN  23. ADMIRE, 24.CONFIDOR \| 25. SPINOACE, 26. SPINTOR  27. KRISOL  28. KARATE  29. ECOTECH  30. XENTARI  31. PERFEKTHION  32. DIBROM  33. DIBROXONE \| 34. PROCLAIM  35. MYCOTRAL  36. SERENADE  37. CLORPYRIFOS  38. TERBUFOS  39. AGROFOS  40. TERBUGRAN  41.CARBOFURAN  42. DIBROM \| 43.TAMARON  44.LORSBAN  45. AGROMIL  46.DIAZINON  47. BASUDIN  48. AMBUSH \| | | | | | | | | | | | | |
|  |  | **PRODUCTO** | Cos1 | Cos2 | Cos3 | Cos4 | **TOTAL** | **PRODUCTO** | Cos1 | Cos2 | Cos3 | Cos4 | | **TOTAL** |
|  |  |  |  |  |  |  |  |  |  |  |  |  |  | |
|  |  |  |  |  |  |  |  |  |  |  |  |  |  | |
|  |  |  |  |  |  |  |  |  |  |  |  |  |  | |

| **PLAGUICIDAS** | | | | | | | |  | | | | | | |
| --- | --- | --- | --- | --- | --- | --- | --- | --- | --- | --- | --- | --- | --- | --- |
| **#** | **20. NOMBRE DE LA PARCELA** | 21. Por favor, dígame la cantidad de cada tipo de plaguicidas que utilizó en esta parcela en los últimos 12 meses. | | | | | |  | | | | | | |
|  |  | \| ***POR FAVOR, UTILIZA LOS CÓDIGOS SIGUIENTES.*** \| \| \| \| \| \| \| --- \| --- \| --- \| --- \| --- \| --- \| \| ***HERBICIDAS***  1. GRAMOXONE*  2. GLIFOSATO*  3. RANGER*  ***FUNGICIDAS***  4. AMBIL*  5. AMISTAR  6. ALTO*  7. CALDO-BORDELÉS* \| 1. BRAVO 2. ROVRAL   10. METALAXYL  11. BELLIS  12. SILBACUR  **INSECTICIDAS**  13. VOLATON  14. SEVIN  15. AVAUNT \| 16. RIENDA  17. TIODAN  18. ENDOSULFAN  19. TIODAN  20. MALATHION  21. LANNATE  22. GUSAFIN  23. ADMIRE, 24.CONFIDOR \| 25. SPINOACE, 26. SPINTOR  27. KRISOL  28. KARATE  29. ECOTECH  30.XENTARI  31. PERFEKTHION  32. DIBROM  33. DIBROXONE \| 34. PROCLAIM  35. MYCOTRAL  36. SERENADE  37.CLORPYRIFOS  38. TERBUFOS  39. AGROFOS  40. TERBUGRAN  41.CARBOFURAN  42. DIBROM \| 43.TAMARON  44.LORSBAN  45. AGROMIL  46.DIAZINON  47. BASUDIN  48. AMBUSH \| | | | | | | | | | | | | |
|  |  | **PRODUCTO** | Cos1 | Cos2 | Cos3 | Cos4 | **TOTAL** | **PRODUCTO** | Cos1 | Cos2 | Cos3 | Cos4 | | **TOTAL** |
|  |  |  |  |  |  |  |  |  |  |  |  |  |  | |
|  |  |  |  |  |  |  |  |  |  |  |  |  |  | |
|  |  |  |  |  |  |  |  |  |  |  |  |  |  | |
|  |  |  |  |  |  |  |  |  |  |  |  |  |  | |

**SECCION D. GASTOS EN ACTIVIDADES AGRÍCOLAS**

| 1. ¿En los últimos 12 meses, cuánto gastó en TOTAL en (…)? | | | | | | |
| --- | --- | --- | --- | --- | --- | --- |
| **ACTIVIDAD** | | Cos1 | Cos2 | Cos3 | Cos4 | **TOTAL** |
| **A.** | Transporte y pago de fletes |  |  |  |  |  |
| **B.** | Almacenamiento y secado de productos |  |  |  |  |  |
| **C.** | Alquiler de maquinaria agrícola |  |  |  |  |  |
| **D.** | Mantenimiento y reparación de maquinaria |  |  |  |  |  |
| **E.** | Alquiler de animales de trabajo |  |  |  |  |  |
| **F.** | Elaboración de subproductos agrícolas o pecuarias |  |  |  |  |  |
| **G.** | Combustibles y lubricantes |  |  |  |  |  |
| **H.** | Alimentos para los animales como: maíz, melaza, sal, concentrados, etc. |  |  |  |  |  |
| **I.** | Vacunas, remedios, medicinas, o productos veterinarios |  |  |  |  |  |
| **J.** | Cercas, comederos, abrevaderos, y ordeñaderos |  |  |  |  |  |
| **K.** | Honorarios por servicios veterinarios |  |  |  |  |  |
| **L.** | Elaboración de subproductos pecuarios |  |  |  |  |  |
| **M.** | 1. Mano de obra agrícola (mozos)   ¿Por cuántos jornales (quetzales/día) contrató usted a esa mano de obra? |  |  |  |  |  |

**SECCION E. ACTIVIDAD PECUARIA**

| 1. ¿Durante los últimos 12 meses, criaron animales como: gallinas, patos, pavos o chompipas, conejos, cerdos, vacunos, etc., en tierras del hogar, en el patio o lote de la casa o en otras tierras? **NO = 0 SÍ = 1** | | | | | | | **CÓDIGO** | | |
| --- | --- | --- | --- | --- | --- | --- | --- | --- | --- |
|  |  |  |  |  |  |  |  | | |
| **NO** | **2. ¿**Qué animales? | NO = 0  SÍ = 1 | **3. ¿**Cuántas (…) tiene ahora? | **4. ¿**En cuánto podría vender uno (…) el día de hoy? | **5. ¿**En los últimos 12 meses, cuántos (….) vendieron en pie (vivos) y a qué precio los vendieron cada uno? | | | **6. ¿**En los últimos 12 meses, cuántos de sus (….) consumieron la familia? | 7. ¿Cuántos compraron en los últimos 12 meses? |
|  |  | **COD.** | **CANTIDAD** | **QUET.** | **CANTIDAD** | **QUET.** | | **CANT.** | **CANT.** |
| **1** | Vacas, toros, terneros |  |  |  |  |  | |  |  |
| **2** | Cabras |  |  |  |  |  | |  |  |
| **3** | Cerdos (coches) |  |  |  |  |  | |  |  |
| **4** | Conejos |  |  |  |  |  | |  |  |
| **5** | Gallinas y pollos |  |  |  |  |  | |  |  |
| **6** | Pavos o chompipas |  |  |  |  |  | |  |  |
| **7** | Patos |  |  |  |  |  | |  |  |
| **8** | Caballos, burros, mulas |  |  |  |  |  | |  |  |
| **9** | Otros, **¿**cuáles? |  |  |  |  |  | |  |  |

**SECCION F. MEDIOAMBIENTE**

| 1. ¿Realiza algunas de las siguientes prácticas en la actualidad? **NO = 0 SÍ = 1** | | | | |  |
| --- | --- | --- | --- | --- | --- |
|  | **COD.** | **PARCELA** |  | **COD.** | **PARCELA** |
| Aboneras |  |  | Uso de semillas nativas |  |  |
| Entierra la basura y echa tierra encima (preparación de la tierra) |  |  | Uso de semillas mejoradas (compradas en agroservicios) |  |  |
| Siembra de vegetación en los cercos |  |  | Siembra de árboles en su parcela agrícola |  |  |
| Conservación de suelos con barreras vivas |  |  | Siembra de frutales en su parcela agrícola |  |  |
| Conservación de suelos con terrazas |  |  | Utiliza Gramoxone para la limpia |  |  |
| Siembra orientada en contra de la pendiente |  |  | Quemas o rozas controladas de los terrenos para la siembra |  |  |
| Reforestaciones |  |  | Manejo de la regeneración natural |  |  |
| Descansa la tierra  ---De ser así, ¿cuánto tiempo le permite descansar? ____________ |  |  | Utiliza la luna para sembrar |  |  |
|  |  |  | Utiliza la luna para tapiscar |  |  |

**PLAGAS**

| 3. ¿Qué cultivos son más afectados por plagas en sus parcelas?   1. Maíz 2. Frijol 3. Brócoli 4. Tomate 5. Papa   ANOTA DE MAYOR/MENOR. | 4. ¿Cuáles son las plagas/enfermedades principales que afectan su terreno de brócoli?   1. Gallina ciega 2. Plutella 3. Gallina ciega y Plutella 4. Otro, ¿cuál? | 5. ¿Ha tenido problemas con el hongo camotillo en su terreno de brócoli? De ser así, ¿en qué parcela, desde cuándo y por qué?  **NO = 0 SÍ=1** | | | | Por favor, por las próximas preguntas, dígame si usted está de acuerdo o no con la frase. **NO = 0 SÍ = 1** | |
| --- | --- | --- | --- | --- | --- | --- | --- |
|  |  |  |  |  |  | 6. Las plagas causan más daño en parcelas de brócoli que en parcelas de maíz o de frijol. | **COD** |
|  |  |  |  |  |  |  |  |
|  |  |  |  |  |  | 7. Hay mas plagas hoy que había hace 5 años . |  |
|  |  |  |  |  |  | 8. Hay insectos que comen las plagas que comen la milpa (que ayudan a combatir plagas). |  |
|  |  |  |  |  |  | 9. Hay insectos que comen las plagas de brócoli  (que ayudan a combatir plagas). |  |
| **CÓDIGO** | **CÓDIGO** | **COD** | **PARCELA** | **AÑO** | **RÁZON** | 10. Las prácticas agrícolas determinan los niveles de plagas. |  |
|  |  |  |  |  |  |  |  |

**SECCION G. INGRESOS Y BIENESTAR**

| 1. En los últimos 12 meses, ¿cómo ha obtenido dinero principalmente, para su familia?  1b. ¿Cuál ha sido la segunda fuente importante de dinero para su hogar?? | | 2. ¿En los últimos 12 meses, cuánto ha ganado su hogar por mes (en promedio)? | 3. En comparación con su hogar, otros miembros de la comunidad están en una posición económico: |
| --- | --- | --- | --- |
| 1. Venta de maíz/frijol 2. Venta de brócoli 3. Venta de papa 4. Venta de cultivos (en general) 5. Venta de animales/productos animales 6. Jornaleo en actividad agrícola **local** (empleado para labores agrícolas) 7. Jornaleo en actividad agrícola **migratorio** 8. Trabajo en la ‘Floristería’ (producción de leatherleaf) 9. Trabajo no agrícola (guardián, trabajador doméstico, etc.) | 1. Trabajo independiente (chofer, carpintero, trabajador casual, etc.) 2. Empleado gubernamental (maestro, salud, etc) 3. Empleado de empresa privada u ONG 4. Venta de artesanías 5. Venta de productos silvestres (mora silvestre, etc.) 6. Pequeño comercio (pequeña escala) 7. Negocios (a mayor escala) 8. Subvenciones para el hogar, beneficios de desempleado 9. Remesas de emigrantes (dentro o fuera del país) | 1. Menos de Q500 2. Entre Q500 – Q1,000 3. Entre Q1,000-Q2,000 4. Entre Q2,000 – Q, 3000 5. Más de Q3, 000 | 1. Mucho mejor 2. Poco mejor 3. Igual 4. Poco peor 5. Mucho peor |
| CÓDIGO | CÓDIGO | CÓDIGO | CÓDIGO |
|  |  |  |  |

| 4. Antes de la introducción de brócoli en Chilascó, cómo era la situación económica en Chilascó:   1. Mucho mejor 2. Un poco mejor 3. Igual 4. Un poco peor 5. Mucho peor | 5. Cómo ve el cambio de las hortalizas en Chilascó?   1. Positivo 2. Negativo 3. Sigue igual | 6. A quien más le beneficia los cultivos de brócoli?   1. El hogar 2. Las empresas 3. El coyote 4. Todas 5. Otra, ¿quién? | Por favor, por las próximas preguntas, dígame si usted está de acuerdo o no con la frase. **NO = 0 SÍ = 1** | |
| --- | --- | --- | --- | --- |
|  |  |  | 7. En Chilascó, hay gran diferencias entre los pobres y los ricos: | **COD** |
|  |  |  |  |  |
|  |  |  | 8. Las empresas de exportación de brócoli se aprovechan de los agricultores |  |
|  |  |  | 9. Las empresas de brócoli no pierden nunca |  |
| **CÓDIGO** | **CÓDIGO** | **CÓDIGO** | 10. Las empresas de brócoli siempre ofrecen precios justos |  |
|  |  |  | 11. El que se arriesga es mejor económicamente |  |

| 12. ¿La siembra de brócoli le ayuda a alimentar a su familia?  ¿Por qué? | | 13. ¿En general, se considera una persona feliz? | 14. ¿Cree usted que puede cambiar el futuro de su vida? | 15. Actualmente, ¿cuáles son sus necesidades prioritarias para mejorar el bienestar de su familia? *Por ejemplo,* las prioridades pueden ser relacionadas a la agricultura, la salud, los gastos y precios, el medioambiente y/o fuentes de empleo.  *REGISTRE LAS TRES PRIMERAS RESPUESTAS..* | | | |
| --- | --- | --- | --- | --- | --- | --- | --- |
| 1. Sí 2. No 3. Sí y No 4. Otro, ¿cuál? | | 1. Muy feliz 2. Feliz en parte 3. Ni feliz ni infeliz 4. Algo infeliz 5. Muy infeliz 6. No estoy seguro/a | 1. Definitivamente sí 2. Probablemente 3. No está seguro 4. Probablemente no 5. Definitivamente no 6. Hay otras personas que tienen el poder | 1. Alimentos para niños pequeños/desnutridos u otros grupos vulnerables 2. Alimentos para el hogar en general 3. Empleo 4. Transferencias de dinero 5. Crédito 6. Aumento de salario/pensión 7. Insumos agrícolas (semillas , fertilizantes, pesticidas) 8. Herramientas agrícolas | | 1. Forraje para animales 2. Riego 3. Agua para uso domestico 4. Servicios de salud (infraestructura, personal) 5. Carreteras 6. Escuela infraestructura/maestros 7. Vivienda/albergue (reparaciones, nuevo) 8. Combustible 9. Seguridad 10. Otra -especifique | |
| **CÓDIGO** | **RAZÓN** | **CÓDIGO** | **CÓDIGO** | **(a) CÓDIGO** | **(b) CÓDIGO** | | **(c) CÓDIGO** |
|  |  |  |  |  |  | |  |
